# Metabolomics Biomarkers of Prostate Cancer: A Systematic Review

**DOI:** 10.3390/diagnostics9010021

**Published:** 2019-02-19

**Authors:** Marouane Kdadra, Sebastian Höckner, Hing Leung, Werner Kremer, Eric Schiffer

**Affiliations:** 1 Numares AG, Am BioPark 9, 93053 Regensburg, Germany; marouane.kdadra@numares.com (M.K.); Sebastian.Hoeckner@numares.com (S.H.); 2 Institute of Cancer Sciences, College of Medical, Veterinary and Life Sciences, University of Glasgow, Glasgow G61 1QH, UK; h.leung@beatson.gla.ac.uk; 3 CRUK Beatson Institute, Bearsden, Glasgow G61 1BD, UK; 4 Institute of Biophysics and Physical Biochemistry, University of Regensburg, 93053 Regensburg, Germany; Werner.Kremer@biologie.uni-regensburg.de

**Keywords:** prostate cancer, metabolomics, biomarkers, systematic review, metabolites, profiling

## Abstract

Prostate cancer (PCa) diagnosis with current biomarkers is difficult and often results in unnecessary invasive procedures as well as over-diagnosis and over-treatment, highlighting the need for novel biomarkers. The aim of this review is to provide a summary of available metabolomics PCa biomarkers, particularly for clinically significant disease. A systematic search was conducted on PubMed for publications from July 2008 to July 2018 in accordance with PRISMA guidelines to report biomarkers with respect to their application in PCa diagnosis, progression, aggressiveness, recurrence, and treatment response. The vast majority of studies report biomarkers with the ability to distinguish malignant from benign prostate tissue with a few studies investigating biomarkers associated with disease progression, treatment response or tumour recurrence. In general, these studies report high dimensional datasets and the number of analysed metabolites often significantly exceeded the number of available samples. Hence, observed multivariate differences between case and control samples in the datasets might potentially also be associated with pre-analytical, technical, statistical and confounding factors. Giving the technical and methodological hurdles, there are nevertheless a number of metabolites and pathways repeatedly reported across various technical approaches, cohorts and sample types that appear to play a predominant role in PCa tumour biology, progression and recurrence.

## 1. Introduction

Prostate cancer (PCa) is the most common cancer in males aged over 70 years and the second most common cause of cancer death in men [1]. Annually, there are more than 1.1 million newly diagnosed patients worldwide. Curative treatment consists primarily of surgery and various forms of radiation therapy [2,3]. Nevertheless, globally each year about 270,000 men die from PCa [4]. Moreover, costs occurring in developed nations in the first year after diagnosis are high. On the one hand, overtreatment of indolent tumours, besides having a negative impact on quality of life, is a major burden on public health care systems. There is a growing body of evidence indicating that an important proportion of patients with high-risk disease may be undertreated, leading to further and often costly treatments for more advanced or metastatic disease [5]. As incidence rates are expected to rise due to demographic changes, improved clinical management of PCa would improve patient outcomes and reduce the cost-burden to health care systems. Most patients with early-stage PCa are asymptomatic [1]. Signs of locally advanced or metastatic disease include unspecific lower urinary tract symptoms that can also arise from benign hyperplasia of the prostatic gland. To date, digital rectal examination (DRE) and testing for prostate specific antigen (PSA) are still the most common clinically used tools for early detection, which however fail to predict clinical behaviour [1]. As PSA has a low specificity and is associated with a high number of false positive rates [1], any nodularity or induration of the prostate gland or increase in PSA-level will consecutively lead to further evaluation by biopsy or via imaging technologies.

While multiparametric magnetic resonance imaging (MRI) holds some potential with continued evaluation to improve prognostic information [6], current clinical practice still relies on histopathological evaluation of biopsy specimens using the Gleason scoring system, which is based on the glandular architecture [1]. Biopsies, albeit the most reliable, remain a problematic assessment of the tumour extent and biology, as the procedure with up to 20 cores taken constitutes a substantial burden and has significant potential side effects and complications. Patients are assigned into clinical risk groups (low, intermediate, or high-risk), depending on PSA-level, Gleason grade and clinical tumour-lymphnode-metastasis (TNM) staging [1]. The extent of PCa may be supplemented with bone scanning, computed tomography (CT) staging or MRI according to clinical risk groups. Unfortunately, this scoring system suffers from a high inter-observer variability [7] and the inherent risk of missing more progressed/aggressive areas of the tumour, leading to misclassification. More importantly, these classifications used for clinical decision-making cannot consider distinct tumour phenotypes and hence fail to reliably predict patients’ individual risk.

The majority of PCa detected by screening have PSA levels between 4–10 ng/mL and moderate Gleason sum scores. These patients are considered to have low to moderate risk disease, and their treatment decisions should ideally be tailored to their anticipated tumour behaviour. “Active surveillance” that defers initial treatment in favour of close monitoring and reassessment of non-aggressive low-Gleason score PCa was introduced. However, it still requires repeated PSA-testing coupled to re-biopsies (often at multiple time points) with additional risks and costs.

Depending on the findings and the psychological distress, clinicians and patients often tend to opt for intense therapy in the absence of any decisive indication for the presence of aggressive disease, which significantly contributes to widespread overtreatment. According to current estimates, up to 50% of PCa patients are subjected eventually to intense therapy, while only 20% are suffering from aggressive disease [1]. Thus, PCa is a common but not particularly aggressive form of cancer.

Once a clinical decision for intense therapy has been made, patients will initially receive surgery or various forms of radiation therapy, which is associated with severe side effects, impacting negatively on quality of life [2,3]. Up to 50% of men treated with surgery or radiation in curative intent will eventually relapse based on evidence of a detectable or rising level of PSA [8]. The current standard of care for metastatic disease is anti-hormonal therapy (+/− docetaxel chemotherapy or second generation androgen receptor pathway inhibitor), as most tumours are hormone-dependent and therefore respond initially well to various forms of androgen deprivation therapy. Unfortunately, most PCas will recur after 18–33 months to develop castration-resistant prostate cancer (CRPC). This cancer subtype requires a multidisciplinary approach and involves counselling of the individual patient to discuss the viable treatment options such as second line hormonal treatment or chemotherapy. Despite ongoing research efforts, including studies with modern tyrosine kinase inhibitors, modern strategies to control CRPC have shown clinical benefits that are not sustained beyond two years [9]. This is mainly due to the fact that stratification of PCa patients based on molecular characteristics has not been implemented, which in turn is explained, at least in part, by the genetic complexity of PCa [10].

All these facts underline the urgent need for novel biomarkers [11] to improve clinical decision making and management of PCa. The rapid advances in molecular technologies allowed identification of various potential biomarkers for PCa. These technologies include genetic sequencing, transcriptomic expression profiling, proteomic and metabolomic profiling. The metabolome represents the complete set of metabolites as end products of cellular processes in a biological cell, tissue, organ or organism. Metabolomics can be considered as a downstream or end-result measure of activities on the level of the genome, epigenome, transcriptome and proteome, and their interactions with the environment [5]. Tumour cells are particularly reported to express distinct metabolic signatures [10] and their interactions with the environment are more and more described as tumour microenvironment and carcinoma-associated fibroblasts that are emerging fields of research [12,13]. Accordingly, over the last decade, an increasing number of studies have attempted to capture metabolomic biomarkers for PCa and these were most recently discussed in several narrative review articles. However, none of these were systematic. Therefore, the aim of this review is to provide a systematic qualitative summary of current evidence relevant to metabolomics biomarkers for prediction, diagnosis, progression, prognosis, or recurrence of PCa according to PRISMA (Preferred Reporting Items for Systematic Reviews and Meta-Analyses) guidelines.

## 2. Methods

This systematic review follows the PRISMA guidelines and is reported in accordance with the PRISMA statement.

### 2.1. Search Strategy

A systematic search was conducted on PubMed for all publications with relation to metabolomics biomarkers of prostate cancer reported from July 2008 to July 2018, using the following combinations of MeSH terms: prostate cancer (tiab) intervention with (metabolite OR metabolomic OR metabolomics) intervention with (marker OR biomarker). Initially, titles and abstracts of all identified studies were screened and reviewed on the basis of the established selection criteria.

### 2.2. Selection Criteria

English articles were selected based on their titles and abstracts for full-text review according to their relevance to the issue of interest. The following inclusion criteria were applied with no restriction to the bio-specimen used: identification of human PCa specific metabolites; indication on PCa diagnosis, prognostics, aggressiveness or recurrence; evidence for the clinical utility of the biomarkers; level of standardization of the analytical platforms used and their limitations. Only metabolomics studies were included; other “omics” results were excluded. In addition, reviews and studies made on animal models of PCa or on cell model systems were excluded. Finally, a screening of the reference lists of included articles resulted in reviewing additional titles and abstracts for potential inclusion.

### 2.3. Data Extraction

The selected studies were thoroughly examined and the following information were extracted from each article: name of first author, year of publication, sample size (specifying the number of cases and controls), analytical platform used, use case, relevant biomarkers candidates, validation of biomarkers, statistical details and relevant comments about the study. Data were independently extracted by two different reviewers (M.K., S.H.) and disagreements regarding the selected information were solved by further review and discussion among them.

## 3. Results

**Figure 1:**
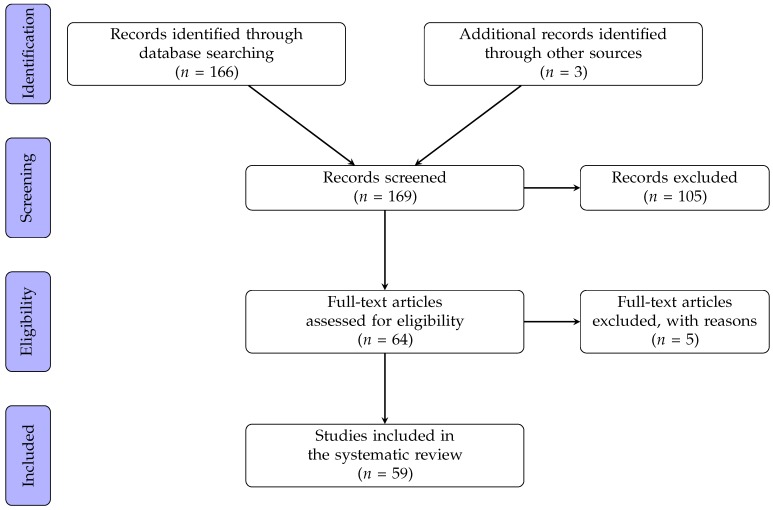
PRISMA flow diagram of the literature search process.

A total of 169 articles were identified in the literature search (Figure 1). The full text was obtained for 64 articles after screening and exclusion on the basis of titles and abstracts. Five of these article were excluded from the analysis after the full text reading. The remaining 59 are summarized in Table 1, Table 2, Table 3 and Table 4.

### 3.1. Study Characteristics

Of all the 59 selected reports, a total of 43 studies were conducted on only one bio specimen as follows: 14 studies were conducted on blood samples, 10 on urine samples and 19 on tissue samples. Eight studies have used more than one of the aforementioned bio specimen in their analysis and thus were reported in more than one table. nine reports analysed other types of biospecimens, such as seminal fluid, or did in vivo magnetic resonance spectroscopy imaging (MRSI), i.e., spectroscopic analysis of the prostate in situ using MRI scanners.

### 3.2. Outcomes

#### 3.2.1. Blood Based Biomarkers

In total, seventeen metabolomics studies were conducted on blood samples (Table 1). Six publications attempted to identify biomarkers of prostate cancer risk. Three of the six studies were nested within the Alpha-Tocopherol, Beta-Carotene Cancer Prevention Study (ATBC) [14,15,16], while one was part of the Prostate, Lung, Colorectal, and Ovarian Cancer Screening Trial (PLCO) [17], another embedded in the JANUS cohort [18] with a further study incorporated within the European Prospective Investigation into Cancer and Nutrition (EPIC) [19]. Ten studies focused on the assessment of biomarkers for diagnostic and/or staging purposes. Only one study focused on the identification of prognostic metabolites. Likewise, only one study reported on biomarkers for therapy prediction was found.

##### Blood Biomarkers Associated with PCa Risk

The ATBC study was a cancer prevention trial that enrolled 29,133 Caucasian male smokers from Finland. These men were aged between 50–69 years at baseline, smoked at least 5 cigarettes per day, and were assigned to one of four distinct intervention groups. The aim of the study was to examine whether the vitamin supplementation with alpha-tocopherol and beta-carotene, either alone or in combination, would prevent lung and other cancers compared to Placebo. All metabolomics studies within the ATBC cohort were conducted by Demetrius Albanes and co-workers with the aim to identify metabolomic profiles associated with the risk of prostate cancer up to 20 years prior to diagnosis. The group applied ultrahigh performance liquid chromatography/mass spectrometry (LC-MS) and gas chromatography/mass spectrometry (GC-MS) for metabolic profiling of fasted serum samples collected at baseline. In their first study, Mondul et al. analysed sera from 148 participants [14]. The case group comprised 74 patients who developed PCa up to 23 years after blood collection, while 74 participants without PCa diagnosis in the same period were selected to match the collection date and age to serve as controls. The authors found a significant (corrected *p* < 0.000119) inverse association of 1 stearoylglycerol with overall prostate cancer risk (Odds ratio 0.34, *p* = 0.00006). Additional biomarker candidates identified included glycerol and alpha-ketoglutarate, but their associations did not reach statistical significance when corrected for multiple comparisons.

In their second study within the ATBC cohort [15], the authors repeated the metabolomics analysis using an additional set of 200 confirmed cases of PCa and 200 controls that were independent of the sample set of the first study. Notably, they were unable to replicate the association of 1-stearoylglycerol and glycerol with increased PCa risk. Additionally none of the detected metabolites achieved statistical significance after correction for multiple testing (*p* = 0.00008). Nevertheless, strong risk associations between molecules involved in energy and lipid metabolism and risk of aggressive cancer were observed. In addition, the previously reported association between alpha-ketoglutarate and risk of aggressive prostate cancer (defined as TNM stage III-IV, AJCC stage ≥ 3, or Gleason ≥ 8) was confirmed with a significance level of *p* = 0.00008 (Odds ratio = 0.69, *p* = 0.02). In addition, the authors identified citrate, a large number of glycerophospholipids, including oleoyl-linoleoyl-glycerophosphoinositol, and long chain fatty acids (LCFA) among the top metabolites associated with risk of aggressive Pca. Most of these biomarkers were inversely related to aggressive PCa risk, with inositol-1-phosphate, a precursor of myo-inositol, showing the strongest association (Odds ratio = 0.56, *p* = 0.002). When stratified by median time from blood collection to diagnosis, distinct metabolites were found to be associated with risk of aggressive PCa. Furthermore, high risk of aggressive Pca was associated with elevated levels of thyroxine and trimethylamine N-oxide (TMAO), a liver metabolite biosynthesised from trimethylamine which in turn is produced from dietary phosphatidylcholines and carnitine by gut bacteria. In contrast, the pyrimidine-nucleoside 2′-deoxyuridine and adenosine 5′-monophosphate, a constituent of ribonucleic acid, appeared related to more indolent forms of PCa.

Using the same sample set, the authors performed a secondary analysis in order to test, whether men diagnosed with ≥ T2 tumours exhibit different metabolite profiles up to 20 years prior to clinical diagnosis [16]. Compared to controls, qualitative differences in metabolite profiles were found for tumour groups and various metabolites were reported; however, most of them did not reach statistical significance after correction for multiple testing. Solely the glycerophospholipid oleoyl-linoleoyl-glycerophosphoinositol already identified in the primary analysis turned out to be significantly associated with subsequent diagnosis of locally advanced (T3) PCa (Odds ratio = 0.49, *p* = 0.000017), but surprisingly not with later diagnosis of T4 disease. Apart from that, non-significantly elevated serum levels of metabolites in histidine metabolism, including the nucleoside 2’-deoxyuridine also found in the primary analysis, and an inverse association with certain glycerophospholipids were found in men with T2 tumours. Men with T3 prostate cancers showed increased sphingolipids and as well as lower glycerophospholipid signals. Men with a later diagnosis of T4 tumour exhibited elevated signals for secondary bile acid lipids, sex steroids and caffeine-related metabolites, while the carboxylic acids fumarate and citrate were decreased in this group compared to controls. Moreover, the authors found consistent lower levels of glycerophospholipids stearoyl-arachidonoyl-glycerophospho-ethanolamine (GPE) and stearoyl-linoleoyl-GPE as well as a positive signal for euricoyl sphingomyelin in T2 and T3, but not in T4 cases. Serum levels of the histidine metabolite 4-imidazoleacetate and the secondary bile acid glycolithocholate sulfate were found to be elevated across all tumour stages.

The same group performed metabolomics profiling using serum samples from the PLCO trial [17], which was a large randomized-controlled trial to evaluate the efficacy of screening methods for different cancers including PCa. Participants were enrolled from 10 centres in the USA between 1993 and 2001 and randomly assigned to either the screening or non-screening arm. From the screening arm, non-fasting serum samples and data on serum total PSA measurement and DRE examination (in contrast to the ATBC studies) were available for analysis. 380 participants with PCa diagnosis during the post-screening trial period, i.e., 4.4–17.0 years after baseline, and 380 controls matched by age, race, study centre, study year, and date of blood collection were included. Numerous metabolites were reported to be associated with PCa risk, but none demonstrated statistical significance following correction for multiple comparisons. They observed an inverse association with overall risk of PCa and risk of aggressive PCa (T3, T4 or Gleason ≥ 8) for several amino acids and their derivatives, while stearoylcarnitine was positively associated. Alpha-tocopherol, primary bile acid, and steroid hormone metabolites were inversely associated with risk of indolent PCa. Positive associations of lipids with overall risk of PCa as well as risk of aggressive PCa were also reported and were inconsistent with findings in the ATBC cohort. Notably, alpha-ketoglutarate and citrate did not replicate and had even opposite regulation in the PLCO cohort. Moreover, previous associations with aggressive PCa observed for e.g., thyroxine and TMAO were not confirmed. However, the association of 2’-deoxyuridine with overall prostate cancer risk could be replicated, as well as the associations of the glycerophospholipid 1-palmitoleoyl-2-linoleoyl-GPC and the bile acid tauro-beta-muricholate.

The JANUS study conducted by de Vogel and colleagues investigated the association of sarcosine and metabolites along the choline oxidation pathway, i.e., from betaine down to serine, with risk of incident PCa [18]. For this study, serum samples were analysed from 317,000 Norwegian men either participating in health screening surveys or blood donation. LC-MS and GC-MS were used for targeted analysis of six distinct metabolites in 3000 serum samples from patients with incident PCa with a mean time to diagnosis of 15.6 years. 3000 control samples were available, being matched by age, date of serum sampling, and county of residence. The results suggested that men with high serum sarcosine (*p* = 0.03) or glycine (*p* = 0.07) levels have modestly reduced PCa risk, whereas serum betaine, dimethylglycine, and serine were not associated with prostate cancer risk. However, the association of sarcosine and glycine only held true when folate concentration was above 13.7 nmol/L. In addition, a high glycine/serine ratio was related to a decreased PCa risk (*p* = 0.001), while other metabolite ratios containing betaine, dimethylglycine, sarcosine or glycine were not.

Schmidt et al. reported various metabolites from several metabolite classes associated with risk of PCa, advanced stage disease and death from PCa using blood samples from the EPIC study [19]. This trial was a European multi-center cohort study aiming to examine how diet is associated with cancer risk. Case group comprised 1077 men diagnosed with PCa after blood collection matched to 1077 controls by the study center, length of follow-up, age, time of day, and fasting status at blood collection. Using a targeted mass spectrometry approach, 122 metabolites were evaluated. Their strongest findings were an inverse association of citrulline with overall risk of prostate cancer diagnosis within the first five years of follow-up, whereas 12 different glycerophospholipids were inversely related to advanced stage disease (TNM stage T3, T4 and/or N1-3 and/or M1). All these associations were significant after correcting for multiple testing. In addition, conventionally significant associations (*p* < 0.05) were seen for three acylcarnitines, two amino acids and a glycerophospholipid with overall risk of prostate cancer, for 29 metabolites including 20 different glycerophospholipids with risk of advanced disease, for 13 metabolites with risk of aggressive PCa (T4 and/or N1-3 and /or M1 or Gleason ≥ 8), and for seven metabolites with death from prostate cancer. However, none of these markers have been validated in an independent sample set.

##### Blood Biomarkers for Diagnosis and Staging of PCa

Andras and colleagues published a study that aimed to identify a metabolomic score for PCa using 90 serum samples from patients who were suspected to have PCa and underwent prostate biopsy [20]. In contrast to most other studies, where specimens were preserved at $-80{\;}^{\circ }C$, serum was stored at $-20{\;}^{\circ }C$. The data set was split into a training/discovery set (*n* = 59) and a validation set (*n* = 31), with 25 patients suffering from benign prostatic hyperplasia (BPH) and 34 patients diagnosed with PCa in the training set and 17 patients with BPH and 14 PCa patients in the validation group. High-performance liquid chromatography coupled to electrospray ionization quadropole Time-of-Flight mass spectrometry (HPLC-ESI-QTOF MS) was performed for a targeted analysis of 18 amino acids and 33 metabolites. No amino acid was significantly different between PCa and BPH patients in the training cohort (*p* < 0.05). However, several of the 33 metabolites were significantly altered, including glycerol-3-phosphate, glycerophosphocholine, distinct lysophosphatidylcholines, retinoic acid, and prostaglandin. Due to a high grade of multicollinearity between metabolites, six metabolites with *p* < 0.02 and the lowest correlations with other compounds were selected for partial least square regression. The resulting score incorporating lysophosphatidylcholine 18:2, homocysteine-inosine, methyladenosine, lipoic acid, hydroxymelatonin and decanoilcarnitine as markers showed an area under the curve (AUC) value of 0.779 (*p* < 0.001) with a sensitivity of 74% and a specificity of 76% for discriminating PCa from BPH in the training set. In the validation set, the score distinguished PCa from BPH with a sensitivity of 88% and specificity of 60%. Including PSA to the metabolomic score did not significantly increase its AUC-value.

Kumar et al. conducted two metabolomics studies using NMR spectroscopy to identify diagnostic PCa biomarkers in serum [21,22]. Both studies lacked independent validation cohorts, but some results of the first report were replicated in their second study. In their first publication [21], the group analysed 102 serum samples obtained from 70 PCa patients (30 harboured high grade (Gleason ≥ 8) and 40 low grade (Gleason ≤ 7) PCa) and 32 healthy controls (HC) after an overnight fast. Bin-based orthogonal projections to latent structures-discriminant analysis (OPLS-DA) models established on 70% of the data as training set. These models yielded accuracies of 90%, 95%, 94%, and 99% for discriminating between HC and PCa patients, HC and low-grade PCa, HC and high-grade PCa, and low grade and high grade cancer, respectively, in the test set (remaining 30% of data). Several statistical approaches, including ANOVA followed by a post hoc Student–Newman–Keuls multiple comparisons test, were applied to identify important metabolites. Alanine, glycine, pyruvate, and sarcosine were found to be statistically significant (*p* < 0.01) and discriminated between HC and PCa samples with an AUC of 0.966 in receiver operating characteristic (ROC) analysis following calculation of discriminant predicted probability scores. While the combination of alanine, glycine, and sarcosine provided the highest AUC (0.970) for differentiating HC and low grade tumours, glycine and sarcosine showed best discriminative power when comparing HC to high-grade tumours (AUC 0.997). Alanine, pyruvate, and glycine were able to distinguish low grade from high grade cancers with an AUC value of 0.978. Unfortunately, it is unclear whether the reported performances in ROC analyses refer to the whole data set or the test set only.

In their second study [22], the same authors profiled 210 fasted serum samples obtained from 65 HC, 70 BPH patients and 75 PCa patients. In contrast to their previous study, proteins and lipoproteins were removed using centrifugal filtration prior to NMR analysis. Univariate analysis (ANOVA followed by Student–Newman–Keuls test) of 52 assigned compounds revealed 13 metabolites that were significantly altered between the three patient groups. These 13 markers were used for multivariate linear discriminant function analysis. Based on this, alanine, sarcosine, and glycine were replicated as markers for discriminating HC from PCa patients and, together with citrate, correctly classified 97% of patients. The same four metabolites in combination with creatinine showed an accuracy of 88% when comparing PCa cases to BPH. Moreover, glycine, sarcosine, alanine, creatine, xanthine, and hypoxanthine appeared important markers to distinguish HC from BPH and PCa patients (86%). Glycine, xanthine, pyruvate, methylhistidine, and creatinine were important metabolites for distinguishing HC from BPH (86%). Metabolomic patterns performed better than clinical parameters (PSA, digital rectal exam, and transrectal ultrasound) in each classification category and consistently showed higher AUC values. When using 75% of the data set as training set and the remaining 25% of samples as test set, categorizations of 83%, 82%, 94% and 89% were achieved in the test set for HC vs. BPH and PCa, HC vs. BPH, HC vs. PCa, and BPH vs. PCa, respectively.

Tessem and co-workers explored the value of combined serum and plasma metabolomics analysis to differentiate 29 PCa patients from 21 control patients with BPH using various techniques [23]. Serum samples were profiled by NMR and GC and identified 28 metabolites, 105 lipoprotein-related variables, and 34 fatty acids. In addition, LC-MS/MS contributed 142 metabolites from plasma samples for analysis. Although several metabolites from the distinct analyses were conventionally significant (*p* < 0.05), none reached statistical significance after correction for multiple testing (Benjamini Hochberg correction, *q* > 0.05). Using OPLS-DA, only data acquired by NMR and MS resulted in significant classification models. A combination of 14 and 12 metabolites from NMR and MS gave best classification results and their discriminative power was assessed in ROC analyses. Among these 26 markers, the carnitine derivatives decanoylcarnitine (C10:0), tetradecenoylcarnitine (C14:1), octanoylcarnitine (C8) as well as the sulfur compound dimethylsulfone, and the amino acids phenylalanine and lysine were all increased in PCa and of high importance for classification. The phosphatidylcholine diacyl C34:4, and lipid signals from (CH2)n -CH2-CH2-CO were increased BPH and also contributed substantially to separation of BPH and PCa groups.

Fan et al. applied random forests to identify metabolite alterations in serum for the detection and the staging of PCa [24]. They reported nine metabolites identified using NMR analysis in serum samples from 42 patients diagnosed with PCa (20 with Gleason 5; 22 with Gleason 7) and 14 men with BPH and PSA follow up for 4–5 years were available. Among the NMR-detected metabolites, only glutamate and formate were significantly elevated in PCa compared to BPH patients (ANOVA, *p* < 0.05). AUC values calculated using random forests with 10-fold cross-validation were 0.876 and 0.532 for distinguishing BPH from PCa and Gleason 5 from Gleason 7, respectively. However, the authors did not mention the most important NMR metabolites for these classifications.

Osl et al. developed a novel feature selection algorithm termed associative voting for identifying biomarker candidates in PCa [25]. Flow injection analysis-MS/MS and LC-MS/MS were applied to serum samples obtained from 114 men screened negative for PCa and 206 patients diagnosed with PCa for targeted analysis of 112 metabolites. Among the PCa patients, 121 men had low grade PCa (Gleason 6) and 85 were diagnosed with high grade (Gleason ≥ 8) PCa. The new algorithm outperformed existing feature selection methods with respect to AUC values in most comparisons of patient groups. For distinguishing PCa from controls, two lysophosphatidylcholines, C16:0 PC and C18:0 PC as well as serotonine, aspartate, and ornithine were top-ranked metabolites. Notably, the two lysophosphatidylcholines, ornithine, and serotonin were selected by at least one of the existing feature selection methods, while aspartate was highly ranked in all three methods tested. No reliable marker candidates were found for discriminating low from high grade tumours.

Zang et al. developed a metabolite-based in vitro diagnostic multivariate index assay (IVDMIA) using serum samples from a cohort of 64 PCa patients and 50 age-matched healthy controls [26]. Metabolomic analysis was performed using UPLC-MS which yielded a total of 480 features. The data set was split into a training (70%) and a test set (30%). An optimum set of 40 discriminative features identified by support vector machine classified PCa patients and healthy controls with an average accuracy of 93% from 10 distinct iterations. Likewise, a good separation between these groups was achieved when principal component analysis as an unsupervised approach was applied to the best 40 features. Additional support vector machine models were built using smaller sub-panels of the 40 metabolites. When only 13 features that were confidently assigned to metabolites were used for modelling, PCa and controls were still distinguished with an accuracy of 85%. Several differential metabolites were identified as fatty acids, amino acids, lysophospholipids, and bile acids.

Dereziński and colleagues performed a targeted analysis of amino acids (proteinogenic and non-proteinogenic) in serum and urine samples to identify potential biomarkers of PCa [27]. By using LC-ESI-MS/MS, they profiled both urine and serum samples from 49 patients diagnosed with PCa and 40 healthy controls without cancer or chronic diseases. In serum, 32 amino acids were detectable in all 89 samples. In univariate analysis, 18 amino acids were significantly different (*p* < 0.05) between serum samples from PCa and those from control patients with 14 amino acids being reduced in PCa patients. These included methionine, ethanolamine, glutamine, isoleucine, arginine, and leucine (all *p* < 0.00002), all of which had AUC values above 0.75 in univariate ROC analyses. Sarcosine (*p* = 0.006), 3-methylhistidine (*p* = 0.008), β-alanine (*p* = 0.013), and aspartate (0.034) were detected at higher levels in PCa patients but showed higher *p*-values and poorer performance in ROC analyses. Methionine, sarcosine, and 3-methylhistidine, together with serine and proline, were the most significant metabolites in multivariate PLS-DA according to their VIP scores. The same metabolites were also most frequently found in models built on two-thirds of the dataset and validated in the remaining third of samples in multivariate ROC analyses based on a Monte Carlo cross-validation approach. In a stepwise discriminant function analysis, sensitivity of 68% and specificity of 100% were observed for classifying PCa and control patients. Among the most important metabolites in serum, ethanolamine and arginine were also reported as potential biomarkers in urine (details see urine section below).

In addition to the above mentioned publications, we found two studies that analysed blood samples but mainly focused on data generated using other biospecimens. In 2009, Sreekumar and colleagues profiled tissue, blood, and urine samples using GC-MS and LC-MS [28]. However, they focused on their tissue data, since they detected most robust differences using this specimen type, and did not report details of their metabolomics plasma profiles (for details see tissue section). The goal of the other report was to discover biomarkers for metastatic PCa [29]. The main work was done using GC-TOF-MS of tissue samples from bone metastases and primary tumours. However, plasma samples derived from patients with high-risk tumours with (M1, *n* = 7) and without bone metastases (M0, *n* = 6) and from patients with benign prostates (*n* = 17) were analysed as well. Using OPLS-DA, ANOVA and Mann–Whitney U-test, seven of 27 identified metabolites were significantly altered, including pseudouridine (an isomer of the nucleoside uridine), creatinine, glucose, glutamate, taurine, phenylalanine, and stearate, the last four of which were also identified as markers of metastatic PCa in bone tissue. In addition, sarcosine was specifically measured using a targeted approach, but no significant differences in sarcosine levels were observed when comparing patients with PCa to patients with benign prostate biopsies or metastatic PCa to non-metastatic PCa.

##### Blood Biomarkers for Therapy Prediction and Prognosis in PCa

The only study focusing on blood biomarkers for predicting treatment response was conducted by Huang and colleagues and published in 2014 [30]. They analysed serum from 18 patients with newly diagnosed PCa (untreated group) and 18 healthy controls (control group) by LC-MS. In addition, serum samples collected from 36 PCa patients who received androgen deprivation therapy (ADT) at the time of CRPC (castration-resistant prostate cancer) diagnosis were examined. Among the treated PCa patients, 18 responded to treatment for less than one year (poor response group) while 18 were sensitive for more than two years (good response group). Based on OPLS-DA, the authors selected 100 and 60 ions showing the largest significant differences (VI *p*-values > 1.5 and *p*< 0.05) between healthy controls and PCa groups and between poor and good responders to ADT, respectively. When comparing these 160 features, 20 ions were common in the two OPLS models and had less than 20% relative standard deviations in each group. Tandem MS identified two bile acids deoxycholic acid and glycochenodeoxycholate, the omega-3 fatty acid docosapentaenoic acid (all involved in cholesterol metabolism), tryptophan, the omega-6 fatty acid arachidonic acid, the nucleotide deoxycytidine triphosphate, and pyridinoline (released from cross-linked collagen fibers during bone loss) as potential biomarkers for predicting response to ADT. All these markers were altered in PCa compared to healthy controls. However, their serum levels in patients who responded to ADT reverted close to the levels observed for the control group. This suggests that serum levels of these metabolites are potential markers for the early response to endocrine therapy.

Like in the field of therapy prediction, only one of the identified studies dealt with prognostic biomarkers in blood [31]. In their work published in 2011, Stabler and colleagues used GC-MS for targeted analysis of metabolites of the methionine metabolism, including sarcosine, dimethylglycine, methionine, homocysteine, cystationine, cysteine, methylmalonic acid and methylcitrate, in serum with the aim to identify markers that predict biochemical recurrence after radical prostatectomy. To this end, they compared pre-surgical serum samples from 30 patients without biochemical recurrence for at least five years after prostatectomy (“recurrence-free”) to pre-surgical sera from 28 patients with biochemical recurrence within two years (“recurrent”). Among the metabolites analysed, only homocysteine, cystathionine, and cysteine were significantly different (*p* < 0.001) between the two populations in Wilcoxon rank sum tests. Each of these metabolites was able to rapidly separate recurrent from recurrence-free patients in Kaplan–Meier curves with cysteine being the most discriminating marker. The same three markers were also independent predictors of recurrence-free survival in Cox proportional hazard regression models. Likewise, these metabolites were the top predictors for recurrence in multiple logistic regression models. The addition of serum homocysteine to a basic model with serum PSA and Gleason score resulted in the greatest improvement in discriminating between recurrence-free and recurrent patients (*p* = 0.0007). The AUC values for models including one of the markers were similar (AUC = 0.86) and superior to the basic model with an AUC of 0.81. Interestingly, when urine samples were analysed, only cysteine along with sarcosine (*p* = 0.03), was found to be significantly different (*p* = 0.007) between the two groups (see below), while homocysteine and cystathionine were not.

#### 3.2.2. Urine Based Biomarkers

Nine studies used urine samples for metabolomics profiling (Table 2). Eight papers reported discriminating metabolites for diagnostic purposes. Two of them also searched for metabolites related to diseases prognosis. Only one study attempted to identify predictive metabolites.

##### Urine Biomarkers for Diagnosis and Prognosis of PCa

Zhang et al. reported urinary alterations associated with PCa validated in a partially independent cohort [32]. They introduced a protocol based on the use of LC-MS with orthogonal hydrophilic interaction (HILIC) and reversed phase (RP) liquid chromatography methodes. The established protocol was used for the analysis of the urinary metabolome and was then evaluated as a diagnostic tool for PCa. Thirty PCa subjects and 30 controls were included. The authors tested different normalization methods (against creatinine levels, osmolality or MS total useful signals/MSTUS) compared to un-normalised data. Orthogonal partial least square discriminant analysis (OPLS-DA) modelling was applied to 25 PCa subjects and 25 controls as a training set while the remaining five cases and controls served as a test set. The discriminative power was higher with creatinine and MSTUS normalisation compared to osmolality and un-normalised data. Thirty additional PCa patients from a different geographic region were compared to the already used control samples using creatinine normalisation. Fourteen metabolites were significantly (*p* < 0.05) altered and four of them were identified as ureido isobutyric acid (an intermediate in the thymine catabolism), indolylacryloyglycine (a compound derived from dietary tryptophan or indole compounds), acetylvanilalinine (a catecholamine metabolite) and 2-oxoglutarate (also called alpha ketoglutarate). The four identified biomarkers had an AUC value of 0.90 which was comparable to the use of the PSA testing (AUC at 0.94). Gkostos et al. measured sarcosine, uracil and kynurenic acid in urine samples of 32 PCa patients prior to radical prostatectomy, 101 patients with increased PSA prior to ultrasonographically guided biopsy. Samples were collected before and after prostatic massage (PM), and 15 healthy volunteers as controls [33]. The objective was to evaluate metabolites as potential biomarkers for PCa detection and progression. The ROC analysis for all participants showed that of the biomarkers, sarcosine (AUC = 0.47) and kynurenic acid (AUC = 0.44) had no diagnostic value. Uracil (AUC = 0.59) showed the highest diagnostic value but without reaching statistical significance (*p* = 0.066). Moreover, none of the metabolites detected in pre-PM showed any diagnostic potential in patients undergoing biopsy. In post-PM urine samples, however, kynurenic acid had a significant diagnostic value (AUC = 0.62). ROC curves were used to also investigate the role of the aforementioned metabolites in the monitoring of PCa progression using Gleason score as a cut-off point between high and low aggression. Results from urine samples of all participants did not show any predictive value for sarcosine, kynurenic acid and uracil (*p* = 0.819, 0.858 and 0.525, respectively).

In a validation study conducted by Gamagedara et al. levels of proline, kynurenine, uracil, and glycerol-3-phosphate were analysed in 126 patients with genitourinary malignancies (PCa and BCa) and were compared to healthy controls (*n* = 68) having no evidence of malignancy (NEM) [34]. The four metabolites did not exhibit any significant differences (*p* > 0.005) when comparing PCa to NEM as well as BCa to PCa and NEM. Furthermore, their urine levels were associated neither with tumour grade nor with tumour stage. However, the biomarkers were highly correlated with urinary creatinine levels, suggesting that their occurrence is mainly regulated by renal excretion. The levels of biomarkers in both cancer and normal samples were not correlated to age or PSA.

Fernández-Peralbo et al. performed an untargeted metabolomics analysis of urine from 62 patients with clinically significant PCa and 42 healthy individuals (both groups confirmed by biopsy) [35]. Twenty-eight significant metabolites were reported (unpaired *t*-test) and used to develop a partial least squares discriminant analysis model characterized by 88% sensitivity and 93% specificity. The stability of the model was assessed in a validation set comprising 30% of the entire samples set not used in the prior training step. Sensitivity and specificity were 63% and 79%, respectively. Several metabolites were found to contribute to clustering of the PCa patients, such as urea and the purine 7-methylguanine. In contrast, amino acids, including tyrosine, citrulline and histidine, together with acetylated and methylated amino acids such as acetyllysine, acetylhistidine, dimethyllysine and trimethyllysine were relevant markers in the control group of individuals with a negative biopsy. In another principal component analysis, imidazole lactate, acetylputrescine and dimethylarginine characterized the healthy group, while a heterogeneous group of metabolites including 5-methyldeoxycytidine-5-phosphate, 7-methylguanosine, acetylcitrulline, acetylaspartatylglutamic acid and acetyltaurine supported clustering of the PCa patients group. Dereziński et al. focused on determining urinary amino acids (proteinogenic and non-proteinogenic) profiles as potential biomarkers [27]. Among the 26 amino acids found to be significantly different between PCa and control groups, γ-amino-n-butyric acid, phosphoethanolamine, ethanolamine, homocitrulline, arginine, δ-hydroxylysine, and asparagine showed the lowest *p*-values (all with *p*< 0.00002) and AUC values above 0.75 in univariate ROC analyses. A stepwise discriminant function analysis using two-thirds of the samples for training showed a sensitivity and specificity in a test set (remaining third of the samples) of 89% and 73%. No significant differences in urinary amino acid profiles between patients with different Gleason scores were observed.

Tanzeela et al. investigated volatile organic compounds (VOCs) emanating from urine samples and their ability to discriminate PCa samples (*n* = 59) from non-cancer ones (*n* = 43) using random forest (RF) and linear discrimination analysis (LDA) [36]. The diagnostic potential of 2,6-dimethyl-7-octen-2-ol, pentanal, 3-octanone, and 2-octanone were analysed and compared to the diagnostic performance of serum PSA using a repeated double cross validation approach. Serum PSA levels alone were able to classify patients with mean accuracies of 61% and 63% using RF and LDA, respectively. Classification based on the four VOCs, yielded similar mean accuracies of 65% and 63%. A combination of PSA levels with urinary VOCs only gave a marginal improvement with accuracies of 71% and 65%, using RF and LDA, respectively. Pérez-Rambla’s study set out to identify potential non-invasive urinary metabolites discriminating PCa from BPH patients using 1H-NMR spectroscopy [37]. For this purpose, an OPLS-DA model was built but did not exhibit any statistical power. After reducing the number of variables (to overcome potential overfitting) and carrying out further analysis, eight metabolites showed statistically significant differences in their urine concentrations between PCa and BPH patients. Urine from PCa patients was characterized by increased concentrations of the branched-chain amino acids valine, leucine and isoleucine, glutamate and pseudouridine, and decreased concentrations of glycine, dimethylglycine, fumarate, and 4-imidazole-acetate. These results were partially consistent with observations made in a study conducted by Struck-Lewicka et al. who used liquid chromatography–mass spectrometry (LC–MS) and gas chromatography–mass spectrometry (GC–MS) for metabolomics profiling [38]. Although Struck-Lewicka and co-workers used healthy individuals (instead of patients with BPH) as control group, they also reported decreased levels of glycine in urine of PCa patients. In addition, glycine, serine, threonine, alanine, isocitrate, aconitate and succinate as well as several carnitines (including dimethylheptanoyl carnitine, propanoylcarnitine, butyrylcarnitine and octanoylcarnitine) were all decreased in urine of prostate cancer patients compared to healthy controls. Stabler and colleagues report metabolites for prediction of biochemical recurrence in urine samples from 54 patients. Of those patients, 25 developed biochemical recurrence within two years and 29 remained recurrence free after prostatectomy [31]. They reported that urinary sarcosine was significantly elevated at the time of surgery in patients who later developed biochemical recurrence. Likewise, urinary cysteine was found to be significantly elevated in biochemically-recurrent patients compared to recurrence-free patients. Urinary dimethylglycine and homocysteine were not significantly different between the two groups. Multiple logistic regression model for prediction of biochemical recurrence was only developed using serum metabolites.

##### Urine Biomarkers for Risk Prediction of PCa

Kosti et al. reported the only pilot study of urinary changes associated with PCa risk prediction [39]. They used a liquid chromatography-tandem mass spectrometry method to determine urinary concentrations of 15 estrogen metabolites in 77 incident PCa cases, 77 healthy controls, and 37 subjects without evidence of prostate cancer based on prostate biopsy. The PCa cases were enrolled prior to initiation of treatment. Univariate analysis revealed that 16-ketoestradiol (16-KE2) and 17-epiestriol (17-epiE3) were significantly lower in PCa cases compared to healthy controls. In addition, 17-epiE3 was also found to be lower among biopsy controls compared to healthy controls (*p* = 0.01). There was an inverse association between the levels of urinary 16-KE2 and 17-epiE3 and prostate cancer risk (*p* trend = 0.02), after adjustment for age, race, smoking status, presence of BPH and time of urine collection. Estrogen concentrations were not affected by body mass index, use of non-steroidal anti-inflammatory drugs, presence of diabetes, family history of prostate cancer or presence of BPH. However, smoking is a modifier of urinary estrogen levels. Men in the lowest quartile of 16-KE2 had a 4.6-fold risk of prostate cancer (Odds ratios = 4.62, 95% confidence interval = 1.34–15.99) compared with those in the highest quartile. However, larger studies are needed to confirm these findings.

#### 3.2.3. Tissue Based Biomarkers

We identified 21 studies that used tissues as biospecimen for the discovery of metabolomic biomarkers of prostate cancer (Table 3). Almost all of them aimed to identify biomarkers for diagnosis and/or staging of PCa: ten publications focused mainly on diagnostic metabolites, while 10 other studies searched predominantly for biomarkers that reflect aggressiveness of the disease. Nevertheless, the diagnostic studies in part also reported on biomarkers for staging and vice versa. Some publications investigated metabolic profiles of genetically distinct subtypes of PCa. In addition, one group aimed to identify biomarkers for predicting biochemical recurrence of PCa, defined as detectable serum PSA level (>0.2 ng/mL) after radical prostatectomy. Three additional studies addressed this issue at least in subgroup analyses. Most studies did paired analysis by comparing cancer tissue with adjacent benign tissue of the same tissue sample and generally used fresh frozen tissue. The majority of studies applied mass-spectrometry approaches on tissue metabolite extracts, while seven studies used high resolution magic angle spinning magnetic resonance spectroscopy (HR-MAS MRS) to analyse intact tissue samples. Only nine of the studies validated their results in independent sample sets.

##### Tissue Biomarkers for Diagnosis of PCa

A relatively stringent validation approach using three independent sample sets was employed by Huan and colleagues, although sample sizes were small [42]. For biomarker discovery, they used a set of 12 non-cancerous tissue samples and 13 PCa tissue samples (10 samples with >30% cancer proportion) obtained ex vivo by core biopsy from 16 subjects after prostatectomy. Metabolite extracts were prepared and the amine/phenol submetabolome was profiled by LC QTOF-MS following 13C/12C dansyl labelling. Of the 4090 metabolites found in total, OPLS-DA and Volcano plotting revealed 52 common metabolites that were significantly different (VIP score of ≥1.5 in the OPLS-DA analysis and fold change of ≥1.5 or ≤0.67 and p-value of ≤0.01) between normal and cancer tissue. Among these, three metabolites, i.e., adenosine monophosphate, spermidine (polyamine interacting with nucleic acids), and uracil, were definitively identified, and were combined with two unidentified metabolites into a diagnostic model based on a linear vector machine approach. The resulting model was initially tested in an independent sample set of 19 PCa and 17 normal tissue samples from 18 subjects. These results were used to further optimise the model by integrating two additional biomarkers that performed well in both the discovery and the first validation set and were putatively identified as phosphorylated ophthalmic acid (a tripeptide and analogue of glutathione) and 2,3-diaminopropionic acid (a non-proteinogenic amino acid). In a second independent validation set consisting of 12 cancer and 12 normal tissue samples from 12 subjects, this optimised model achieved 85% sensitivity and 91% specificity for classifying normal and cancer tissue samples.

In another study that had a validation set available, transcriptomics and metabolomics were combined to identify altered metabolic pathways in prostate cancer [43]. By this “dual omics” approach, significant changes in cysteine and methionine metabolism, nicotinamide adenine dinucleotide (NAD) metabolism, and hexosamine biosynthesis were found. For metabolomic analysis, LC-MS was applied on tissue extracts from 25 paired PCa and adjacent benign tissue samples for biomarker discovery and from 51 PCa and 16 BPH patients for validation. The authors aimed to identify metabolites that are significantly (*p* < 0.05) correlated with disease progression but unaltered in normal tissue or BPH. Their strongest finding was significantly elevated sphingosine levels in both discovery and validation cohorts (*p* < 0.001). Sphingosine increased with disease progression and showed an AUC of 0.81 in the validation cohort. Besides sphingosine, citicoline (an intermediate in phosphatidylcholine synthesis and also known as cytidine diphosphate choline), choline, pantothenic acid, carnitine C4-OH, GPC, NAD (all increased in PCa), phenylacetyl-glutamine and carnitine C14:2 (both decreased in PCa) showed significant correlation with PCa. In a study comparing different biospecimens, Sreekumar and colleagues performed metabolomic analysis on tissue and matched urine and plasma samples [28]. They used GC-MS as well as LC-MS for metabolomic profiling and found the most robust differences between PCa and non-PCa when using tissue extracts derived from biopsy samples as biospecimens. They thus focused on the tissue datasets, where 16 benign tissue samples were compared with 12 localized PCa and 14 metastatic PCa samples using Wilcoxon rank-sum test (*p* < 0.05). They identified 37 and 91 known metabolites that were altered in localized PCa compared to benign tissue and in metastatic PCa compared to localized PCa, respectively. The authors highlighted six metabolites, namely sarcosine, uracil, kynurenine, glycerol-3-phosphate, leucine, and proline, which were reported to be significantly and consistently increased from benign tissue over localized to metastatic PCa. When using a support vector machine classification algorithm to discriminate between these groups these markers exhibited *p*-values <0.001 in 100% of the leave-one-out cross-validated datasets.

These metabolites (except for sarcosine) were recapitulated by two subsequent studies (see below), one that focused on biomarkers for PCa aggressiveness [44], and one that developed a method for performing metabolomics and histopathology on the same sample [45]. Sreekumar and co-workers focused on sarcosine as potential biomarker for PCa and were able to confirm the significant increase of sarcosine with disease progression and its differential behavior in an independent set of 25 benign, 36 localized PCa and 28 metastatic PCa samples. In urine samples from 44 biopsy-positive and 51 biopsy-negative patients, sarcosine exhibited AUC values of 0.71 and 0.67 in ROC analyses for urine sediments and supernatants, respectively. The role of sarcosine in PCa was examined further using extensive cell culture experiments. In 2015, Liu and colleagues analysed the metabolomic dataset established by Sreekumar et al. using a novel approach, directed random walk on global gene-metabolite pathway graph (DRW-GM), for joint analysis of the metabolomic profiles together with matched gene expression profiles [46]. By evaluating the topological importance of genes on a reconstructed gene-metabolite graph and by applying logistic regression to construct classifiers, they were able to discriminate between benign and localized PCa tissue and between localized and metastatic PCa tissue with AUC values of >0.85 in the training data set as well as in three independent data sets. Amongst others, proline and sarcosine were found to be topologically important differential metabolites for differentiating localized from metastatic PCa, replicating these metabolites as already identified by Sreekumar and colleagues. Besides proline and sarcosine, the polyamines spermidine, spermine and putrescine as well as 4-Acetamidobutanoate (a derivative of gamma-Aminobutyric acid) were frequently selected metabolites for discriminating localized from metastatic PCa, while glycine was important for distinguishing benign vs. localized PCa. The central aim of the study from Shuster and colleagues was to describe a method that allows metabolomic and histological analysis on the same sample [45]. They fixed 96 biopsy specimens obtained from eight prostatectomies in 80% methanol:water to extract metabolites and demonstrated that subsequent processing of the tissue for staining with hematoxylin and eosin was successful. They also performed GC-MS and LC-MS/MS on extracts from 14 benign and 14 PCa-positive biopsies and detected significantly (*p* < 0.05) changed levels for 83 metabolites in cancer-containing tissue. Highest increases in cancer biopsy extracts were observed for cysteine, the unsatturated fatty acids dihomo-linoleate and docosapentaenoate, N-acetylaspartate, N-acetylglucosamine (glucose derivative and constituent of hyaluronic aicd), uracil, xanthine, and the glycerophospholipid 1-stearoylglycerophophoinositol. In addition, they evaluated the behavior of the set of six metabolites described by Sreekumar et al. (except for sarcosine which was below the detection limit) and were able to recapitulate the previous findings, i.e., higher levels in cancer compared to benign tissue extracts and in T3 PCa compared to T2 PCa. Moreover, they assessed the levels of known biomarkers reported from in vivo studies using MRSI for assessing the metabolomic profile of PCa in situ, i.e., within living patients. Consistent with the in vivo data, choline, lactate, and alanine were increased in cancer compared benign tissue and at least choline was higher in T3 cancer than in T2 cancer. Citrate and the polyamines putrescine, spermidine, and spermine exhibited more complex behavior, as these metabolites were detected at increased levels in T2 but at lower concentrations in T3 PCa when compared to benign tissue samples.

In another study lacking a validation cohort, a German group around Glen Kristiansen used GC-MS and LC-MS/MS to analyse tissue extracts of PCa and matched non-cancerous adjacent tissue obtained via punch-biopsy from cryo-sections of 95 prostatectomies [47]. Among the 124 differential metabolites identified (FDR-corrected *p*-value < 0.05), 12 known metabolites overlapped with differentiating metabolites discovered in both the studies of Sreekumar et al. and Shuster et al. (see above ), namely 2-aminoadipate (an intermediate in lysine metabolism), alanine, cysteine, glutamate, glycine, histidine, leucine, malate, proline, threonine, uracil, uridine. Based on four distinct criteria, the authors selected eight metabolites exclusively found in their study as well as 2-aminoadipate as common metabolite for further evaluation. Among these, the long-chain fatty acid tricosanoic acid showed the highest AUC value for discriminating malignant from non-malignant tissue in ROC analysis (AUC of 0.86), which was only slightly below the AUC value of 0.88 when all nine metabolites were combined. AUC values > 0.8 were also reported for other long chain fatty acids namely cerebronic acid and 2-hydroxybehenic acid. None of the biomarkers were able to distinguish between Gleason score <7 and ≥7, and only gluconic acid showed moderate discriminating power to differentiate between pT2 and pT3 tumours (AUC of 0.64).

The group around Massimo Loda and colleagues conducted two metabolic studies using GC-MS and LC-MS/MS. In their first study, they investigated whether prostate cancer subtypes driven by distinct oncogenes, namely AKT1 or MYC, exhibit distinct metabolic profiles. Untargeted metabolomic profiling was done in cell line and prostate samples from mice and humans, while selected metabolites, including the fatty acids oleic acid, arachidonic acid and docosahexaenoic acids as well as 2-aminoadipate and creatine, were validated in human tissue [48]. While the latter two metabolites were increased in AKT1-driven tumours, oleic acid (*p* < 0.01), arachidonic acid (*p*< 0.05), and docosahexaenoic acid (*p*< 0.05) were found significantly increased in MYC-driven tumours. The main goal of their second study was to evaluate whether formalin fixed paraffin embedded (FFPE) tissue specimens can be used for metabolic profiling and the results were compared to metabolomics performed on fresh frozen tissue [49]. Besides work on cell lines, metabolite extracts from twelve human paired tissue samples obtained by punch biopsy from tissue slices from radical prostatectomies were analysed. Eight of the paired tissue samples were used as training set and 32 metabolites were found to be significantly different (*p*< 0.05) between PCa and benign samples in both frozen and FFPE tissue. OSC-PLS modelling was performed and correctly discriminated between normal and cancer tissue in the validation set (four matched tissue pairs). The relevant metabolites were however not disclosed.

In addition to the above mentioned studies, there were two studies with small sample sizes. Wang and colleagues used a MALDI-FTICR-MS approach to profile three matched, intact tissue pairs and identified differential metabolites using student’s *t*-test [50]. However, they did not highlight or evaluate further any of the detected metabolites. Likewise, Brown et al. applied GC-MS and LC-MS/MS on tissue extracts from eight matched pairs of cancer and non-cancerous adjacent tissue samples, but did not evaluate further the 40 metabolites found by Welch’s two sample *t*-test and hierarchical clustering [51].

##### Tissue Biomarkers for Staging of PCa

Among the identified metabolomic studies focusing on metabolite biomarkers for staging PCa, four studies were conducted by M.B. Tessem’s group from Norway using HR-MAS-MRS for metabolomic analysis of intact tissue. In one of their first studies, the group aimed to discover metabolite biomarkers for aggressive PCa by metabolomics profiling [52]. They included 158 tissue samples from 48 snap-frozen tissue slices taken from 48 prostatectomies. Of these samples, 47 were normal prostate tissue from non-cancerous adjacent areas while 111 samples contained cancer tissue with 81 defined as high grade (Gleason ≥ 7). They used 80% of the sample set for biomarker discovery and tested on the 20% remaining samples, with repeating this procedure 20 times with different randomly chosen training and test sets. Using PLS-DA approaches, models were obtained that correctly classified cancer and benign tissue with average sensitivity of 87% at a specificity of 85% (*p* < 0.001). Relevant metabolites were citrate, taurine and creatine (all decreased in PCa samples) and glycerophosphocholine, phosphocholine, choline, and glycine (all increased in PCa samples). Correct classification of normal, high grade and low grade PCa by PLS-DA was achieved with overall accuracies of 86%, 77%, and 66%, respectively. Absolute quantification of metabolite levels revealed significant differences between low and high-grade cancer for spermine and citrate (Benjamini–Hochberg corrected *p*-values < 0.05), while choline metabolite levels did not change significantly with Gleason score. Thus, spermine and citrate contribute mainly to the observed higher total choline+creatine+polyamines over citrate (CCP/C) ratio in high grade than in low grade tumours. Interestingly, none of the metabolites distinguished Gleason score 7 cancers from cancers with higher scores.

In another study published in 2013, Selnæs et al. assessed 40 tissue samples from prostatectomies of 13 patients that underwent MRI prior to surgery and compared the ex vivo spectra to the in vivo spectra recorded during MRI at the same location [53]. They calculated the choline, creatine and spermine over citrate ratio (CCS/C) and found increased CCS/C ratios with increasing Gleason score by Spearman’s rank analyses (Spearman’s r = 0.69, *p* < 0.001). When comparing non-cancer, low risk (Gleason < 3 + 4), and high risk (Gleason > 4 + 3) groups, significant differences were also observed in the CCS/C ratio (*p* < 0.05). In a more recent study [54], the group used a discovery cohort of 129 (95 cancer and 34 benign) tissue samples from 41 patients to examine whether non-canonical Wnt pathway (NCWP) and epithelial-to-mesenchymal transition (EMT) are associated with metabolic alterations and aggressiveness in prostate cancer. Besides gene expression analysis, the authors conducted a targeted analysis of 23 metabolites in tumour subgroups defined as having low, intermediate or high activation of NCWP-EMT based on the newly identified gene expression signature. Spermine and citrate were found to be significantly decreased in samples with high NCWP-EMT score compared to samples with low or intermediate NCWP-EMT score (Benjamini Hochberg-corrected *p* < 0.05). Levels of taurine (a non-proteinogenic sulfur amino acid and, amongst other, inhibitory neurotransmitter) and phophoethanolamine (precursor molecule for glycerophospholipid and sphingomyelin derivatives) were significantly altered between low and intermediate score groups (*p* < 0.05). Since the NCWP-EMT score was reported to be associated with biological recurrence and metastasis, spermine and citrate may therefore be considered indirectly as biomarkers for aggressive PCa. In 2016, Tessem et al. used the same sample set to assess the metabolic profile of PCa driven by the TMPRSS2-ERG gene fusion [55], which is associated with higher risk of progression and aggressiveness of disease. Besides transcriptomic profiling, they again performed a targeted analysis of 23 metabolites. Among the cancer samples, 34 samples were classified as having high levels of ERG (ERGhigh), while 30 and 31 samples were classified as ERGlow and ERGintermediate, respectively. Levels of ethanolamine, phophocholine and phophoethanolamine increased with ERG status. Compared to ERGlow, ERGhigh tumours showed significantly (*p* < 0.05) decreased levels of citrate, spermine, putrescine and glucose, while glycine levels were significantly increased. These trends held true only for spermine and citrate in the validation cohort. However, after correction for multiple testing (Benjamini–Hochberg correction), the difference in spermine and citrate concentrations remained statistically significant only in the main cohort (*p* < 0.001).

Besides their publication on diagnostic biomarkers, Kristiansen and co-workers focused on PCa staging in subsequent studies [56,57]. In 2013, the group conducted a targeted analysis of sarcosine to evaluate its role as a biomarker for aggressive disease and disease progression [56]. To this end, extracts were prepared from paired PCa and adjacent benign tissue samples from 92 prostatectomies and analysed by GC-MS. Although normalized sarcosine levels (normalized to the weight of each sample and to the median of reference samples) were slightly (7%) but significantly (*p* < 0.05) higher in PCa than in benign tissue, no significant differences were observed between tumour stage (pT2 vs. pT3) or grade (neither Gleason <7 vs. Gleason ≥7 nor Gleason ≤7 vs. ≥8). Consistently, sarcosine showed a moderate AUC value of 0.59 for discriminating PCa and benign tissue, but inferior performance for predicting tumour grade or stage (AUC 0.54). Moreover, no association of sarcosine with biochemical recurrence was found, leading the authors to argue against sarcosine as a suitable marker for tumour aggressiveness or biochemical recurrence. In a more recent study based on an untargeted approach, Meller et al. profiled tissue metabolite extracts from matched tumour and normal adjacent tissue taken from 106 prostate specimens after prostatectomy using GC-MS and LC-MS/MS [57]. ANOVA analysis (*p* < 0.05) confirmed the previously found association of gluconic acid with Gleason score [47]. Besides gluconic acid and amongst others, pantothenic acid was positively associated with Gleason score, while maltose, fructose-6-phosphate, and cholesterol were negatively correlated. Like Hansen et al. in their work from 2016 [55], they additionally analysed the metabolic profile of prostate cancers with ERG translocation. Compared to ERG negative cancers, differential metabolites included gluconic acid and maltotriose, both being negatively associated with the presence of ERG translocation, as well as the long chain fatty acids cerebronic acid, 2-hydroxybehenic acid and tricosanoic acid that were all positively correlated with ERG translocation. Citrate, spermine and putrescine were significantly decreased in ERG-positive samples. McDunn et al. analysed two cohorts with a total of 331 PCa tissue samples and 178 benign tissue (matched to 178 of the PCa specimens) samples using GC-MS and LC-MS/MS without leaving a subset of samples for validation purposes [44]. Tissue samples derived from frozen optimal cutting temperature (OCT)-embedded tissue from radical prostatectomies and metabolites were extracted for metabolomic analysis. When comparing benign with PCa tissue, almost all significantly altered metabolites found by Sreekumar et al. (see above) replicated in this study (*p* < 0.05 corrected using the Benjamini–Hochberg method), including the progression-associated metabolites glycerol-3-phosphate, kynurenine, proline, threonine, and uracil. Notably, sarcosine was only significantly elevated in tissue samples with Gleason score of 8 or higher. Besides these findings, the authors mainly focused on metabolites that are correlated with aggressive PCa with extracapsular extension, Gleason pattern progression and tumour spread into seminal vesicles and regional lymph nodes as clinically defined endpoints. Calculation of the odds ratios for the distinct endpoints revealed NAD+, N-acetylaspartate, putrescine, glucose as top markers (highest and lowest odds ratios) for extracapsular extension, while choline phosphate, glycerol-3-phosphate, putrescine, 6-sialyl-N-acetyllactosamine (oligosaccharide involved in immune response regulation and colon cancer progression) showed highest associations with tumour spread. Proline, malate, ADP-ribose (occurs as post-translational modification of proteins and is involved in cell signaling, DNA repair and gene regulation) and 6-sialyl-N-acetyllactosamine were top ranked metabolites associated with Gleason pattern progression. Interestingly, when looking for metabolites for improving clinical performance of nomograms, different sets of metabolites turned out to be important for predicting organ confinement when added to the Partin table (5,6-dihydrouracil, choline phosphate, glycerol, and methylpalmitate (fatty acid methyl ester)) and for predicting 5-year recurrence when added to the Han table (7-ahydroxy-3-oxo-4-cholestenoate (involved in primary bile acid biosynthesis), pregnen-diol disulfate (steroid sulfate oxoanion), and mannosyl tryptophan(glycopeptide)).

In 2011, Keshari et al. published a targeted analysis of phospholipid metabolites in prostate cancer [58]. 1D and 2D HR-MAS-MRS were applied to analyse 49 tissue samples obtained via core biopsy following prostatectomy. The tissue samples consisted of 13 high grade, 22 low grade, and 14 benign prostate tissues. Consistent with the previous findings of Tessem and colleagues (see above), citrate and polyamines were found to be reduced in PCa tissue. In addition, an increase in choline, phosphocholine (PC), glycerophosphocholine (GPC), phosphoethanolamine (PE) and glycerophosphoethanolamine (GPE) levels was observed compared to benign tissue (*p* < 0.05). Moreover, PE and GPE increased especially from benign to low grade PCa, while concentrations of PC and GPC showed the highest increase between low grade and high grade PCa. Interestingly, these findings contrast to the results published by Tessem et al. [52], which did not observe any significant differences in choline- or ethanolamine-containing metabolites between low and high grade cancers.

The aim of a more recent study of the same group published in 2014 was to identify metabolomic biomarkers that are correlated with Ki-67 staining and pathologic grade in histopathology and are able to discriminate between aggressive and indolent cancer recurrences in patients who received radiation therapy [59]. To this end, HR-MAS-MRS was applied to 124 biopsy tissues, 71 samples from 47 men with untreated prostate cancer and 53 samples from 39 men who received radiation therapy (average 6.4 years before biopsy). The majority of these biopsy specimens represented benign tissue (58 and 32 for untreated and treated patients, respectively), while 5 and 7 were indolent and 8 and 12 were aggressive cancer (untreated and treated PCa, respectively). A significant correlation (*p* < 0.05) with Ki-67 staining, which is increased in more aggressive cancer, was observed for phosphocholine (PC), glycerophosphocholine (GPC), and free choline. In untreated cancer, levels of choline, PC, GPC, glutamate, alanine, and lactate were significantly (*p* < 0.05) increased between indolent and aggressive cancer. The same was true for benign vs. indolent cancer tissue. In contrast, the concentrations of citrate and polyamines were significantly lower in cancer compared to benign tissue. Although signal intensities were reduced compared to samples from untreated prostates, there was an increase in choline, PC, and GPC from benign tissue over indolent to aggressive cancer in tissue from treated prostates. Moreover, these metabolites were significantly higher in aggressive cancer tissue than in indolent cancer (or benign) tissue. Lactate levels were also significantly elevated in post-radiation cancers but were unable to distinguish between indolent and aggressive cancer. The ratio of total choline, i.e., choline + PC + GPC, to creatine ratio was significantly higher in aggressive vs. indolent cancer and predicted aggressive recurrent prostate cancer after radiation therapy with an AUC value of 0.95 in ROC analysis.

In 2010, Thysell and colleagues published a metabolomics study that aimed to identify metabolite biomarkers for metastatic PCa using primarily tissue from bone metastases but also primary tumour and plasma samples [29]. Following GC-TOFMS of tissue extracts from bone metastases (*n* = 14 samples) and adjacent normal appearing bone tissue (*n* = 10), they used OPLS-DA, ANOVA and Mann–Whitney U-test to discriminate between the groups and 34 known of 71 significantly differential metabolites (VIP > 0.9 (OPLS-DA) or *p* < 0.05 (Mann–Whitney-U)) were identified. The resulting OPLS-DA model was able to discriminate PCa bone metastasis samples (*n* = 6) from normal bone samples (*n* = 11) in an independent cohort. When modelling was repeated using the test set, most metabolites identified in the discovery set were replicated as significantly separating biomarkers. Moreover, bone metastases from PCa were distinguishable from bone metastases of other cancer in OPLS-DA. Besides several amino acids, myo-inositol-1-phosphate, citrate, fumarate, glycerol-3-phosphate, and fatty acids, especially cholesterol was found to be the most important differential marker. The authors also applied metabolomic analysis to primary high-risk tumour samples with and without bone metastases (M1, *n* = 7 and M0, *n* = 6) and benign prostate samples (*n* = 17). Eight of 13 known metabolites were found to be significantly altered both between M1 and M0 and between M1 and benign, namely malate, dehydroascorbic acid (oxidized derivative of ascorbic acid o vitamin C), urea, hypoxanthine, asparagine, threonine, fumarate, and linoleic acid (the last four were also found in bone metastases samples). Since sarcosine was not detected by profiling, this metabolite was specifically measured using a targeted approach. Sarcosine was increased in PCa bone metastases compared to normal bone tissue, but seemed to be unaltered between bone metastases of different origins and between benign prostate and primary PCa tissue.

##### Tissue Biomarkers for Staging of PCa

Only one study performed metabolomic profiling in tissue with the main goal to find metabolite biomarkers that predict the risk of biochemical cancer recurrence (detectable serum PSA > 0.2 ng/mL). However, Kristiansen and colleagues at least addressed this issue in subgroup analyses in their two studies on PCa diagnosis [47] and staging [57]. In 2010, Maxeiner et al. published a retrospective study of prostatectomy cases from which needle biopsies had been analysed by HR-MAS-MRS [60]. They matched 16 PCa cases with biochemical recurrence during follow-up with two distinct control sets randomly selected from 183 patients without biological relapse. Matching was done by age, Gleason score, and observation period and was based on either clinical (first control set) or pathological stages (second control set). Given the low number of cases with biochemical cancer recurrence, these two control groups were used to create two distinct data sets, which however shared the identical 16 recurrence cases, for biomarker discovery and testing. The authors performed principal component analysis and canonical analysis of the first nine principal components and four principal components correlated with pathological findings on the clinical-stage-matched dataset for biomarker discovery. The resulting metabolomic profiles were then applied to the second dataset comprising the pathological-stage-matched controls and yielded overall accuracies of 71% (four components) to 78% (nine components). Spermine, glutamine, the inositol stereo-isomers myo-inositol and scyllo-inositol, phophoryl choline and glutamate were reported to be the major discriminating metabolites in these profiles.

In their studies on metabolite biomarkers for PCa diagnosis and staging (see above), Kristiansen and colleagues also evaluated their biomarkers for prognostic potential to predict biological recurrence. In the diagnostic study [47], they analysed their selected set of nine metabolites in Kaplan–Meier and Cox regression analyses, in which aminoadipic acid, gluconic acid and the trisaccharide maltotriose turned out to be associated with biological relapse (*p* < 0.05 corrected by Benjamini–Hochberg method). However, when combined with tumour stage and Gleason score in multivariate Cox regression analysis, only aminoadipic acid was an independent marker for recurrence risk prediction. This metabolite set pre-selected for diagnostic purposes did not include any of the biomarkers found by Maxeiner et al., and replication of results was thus not possible. In their second study focusing on biomarkers for PCa staging [57], the same group looked for prognostic biomarkers without a pre-selection step using 254 candidates. In Cox Hazard Ratio analysis, nine amino acids or amino acid derivatives were among the top ten metabolites (*p* < 0.05 corrected by Benjamini–Hochberg method). However, none of the markers found by Maxeiner et al. could be replicated. Tryptophan and tyrosine showed the highest prognostic potential for predicting biochemical relapse (with *p* < 0.0001). In their targeted analysis of sarcosine as PCa marker, they found no evidence for sarcosine levels being correlated with biochemical recurrence [56].

#### 3.2.4. Other Specimen Types

Besides the work on PCa biomarkers in blood, urine, and tissue, literature search revealed nine additional publications that were either performed on clinically rarely used bio specimens or were conducted in vivo using MRI-based spectroscopy of prostates in situ (Table 4). Two studies used urine extracellular vehicles (EVs) in an attempt to increase the efficiency of biomarker discovery and one study applied NMR-based metabolomics to seminal fluid. All of the six remaining reports presented data from in vivo MRSI studies and assessed their relationship to PCa diagnosis or aggressiveness.

##### PCa Biomarkers in Urine Extracellular Vesicles

Extracellular vesicles (EVs) are small particles with a lipid bilayer. EVs are released from cells by outward budding from the plasma membrane or by fusion of multivesicular bodies with the plasma membrane or after apoptosis. They typically contain a mixture of cytoplasmatic proteins, lipids, and RNA representative to their cellular origin. Clos-Garcia et al. aimed at the identification of metabolic alterations detectable in urine EVs associated with PCa cancer pathogenesis and progression. To this end, they compared metabolic profiles of EVs in PCa vs. BPH, PCa pathological TNM stage 3 vs. 2 and finally perineural invasion Pn1 vs. Pn0 within the T2 group [61]. EVs were isolated by differential ultracentrifugation from urine and analysed by UPLC-MS metabolomics analysis. The number of EVs per mL did not differ between different groups; however, their size increased with tumour stage, meaning that major differences of EV particle sizes were observed between BPH and T3. Univariate analysis revealed a higher abundance of phosphatidylcholines (PC) in BPH samples and acyl carnitines and sterols were more abundant in PCa samples. Ceramides with low carbon number in their acyl chains were increased in PCa samples in contrast to those with higher carbon number (>C23). Moreover, carboxylic acids and glycerolipids were slightly decreased, and vitamins were increased in PCa EVs. As for the fatty acid family, arachidonic acid (20:4n-6) was decreased in PCa samples while other polyunsaturated fatty acids with shorter carbon chain (16:3) were significantly increased in the PCa group. When searching for biomarkers for staging of PCa, three ceramides [Cer(d18:1/16:0), Cer(d18:1/20:0), Cer(d18:1/22:0)] one glycerophospholipid [PC(30:0)], and one stearoylcarnitine [AC(18:0)] showed significant differences (*p* = 0.031, 0.049, 0.040, 0.052 and 0.028, respectively) between T2 and T3 subgroups. Finally, they also tried to identify metabolites associated with disease prognosis. EV samples with perineural invasion (Pn1) had significantly lower abundance of cyclic AMP (cAMP) and higher abundance of the combination of isomers of the steroid hormone metabolites androsterone sulphate and etiocholanolone sulphate. However, sample size for this purpose was low and the unsupervised multivariate analysis was unable to separate any of the above-mentioned groups.

In a similar context, Puhka et al. also conducted a proof-of-concept study of PCa-derived changes in urinary EV (uEV) [62]. Their focus was on the detection of metabolites that were altered in pre-prostatectomy cancer samples relative to healthy controls and post-prostatectomy samples. Several normalization methods were tested: normalization to EV volume, EV number, CD9 optical density, other metabolites, and urine volume or urine creatinine. Normalization to EV volume, number and CD9 optical density yielded similar results showing lower levels of adenosine, glucuronate, isobutyryl-L-carnitine, and D-ribose 5-phosphate (nucleotide precursor) in uEV samples before prostatectomy compared to control uEV samples and after prostatectomy. In particular, glucuronate exhibited the largest difference with all three normalization methods. In addition to the aforementioned metabolites, normalization to EV-derived factors resulted in lower levels of methylhistamine, creatine, glutathione, NAD+ and two carnitines propionylcarnitine and isovalerylcarnitine. Three of the previously identified metabolites, namely glucuronate (to choline, hippurate and niacinamide ratios), isobutyryl-L-carnitine (to NAD+ or carnitine ratios) and D-ribose 5-phosphate (to niacinamide ratio), were also low in the pre-prostatectomy uEV samples when normalised to other metabolites. A lowered ratio of 1-methylhistamine to choline in the pre-prostatectomy samples compared to all the other groups (each *p* < 0.01) was also observed. Normalization to choline revealed low levels of other metabolites in the pre-prostatectomy samples among which ratios of guanidoacetic acid (intermediate of the urea cycle), taurine and isovalerylcarnitine to choline were statistically significant. Since robust discrimination of pre-prostatectomy uEV samples from the other study groups was not possible after normalisation to urine volume or creatinine levels, the authors concluded that normalisation to EV-derived parameters or analysis of metabolite ratios should be the preferred for identifying cancer-related alterations in uEV metabolites profiles.

##### Seminal Plasma

Roberts et al. conducted a study using seminal plasma for selection and monitoring of active surveillance candidates using NMR based metabolomics [63]. One hundred fifty-one men who were investigated for elevated PSA and/or abnormal DRE were included in the prospective cohort study. Ejaculate specimens were obtained prior to or at least one month after prostate biopsy and NMR spectra were obtained. Glucose signals were most dominant in most samples and their influence on the analysis was unpredictable. Therefore, these signals were excluded from further analysis. Risk stratification (low, intermediate, high risk) was performed according to the D’Amico criteria [64]. Among 151 subjects, PCa status (positive = 98, negative = 53) and D’Amico risk (high = 82, low = 69) were used as dependent variables for logistic regression analysis. Unsupervised multivariate statistical analysis using PCA analysis did not show any clustering into clinical groups. However, supervised partial least squares analysis, using the presence of clinically significant PCa according to the D’Amico criteria (csPCa) as the predictive variable, showed that lipids/ lipoproteins are associated with variation for csPCa. A subgroup of 11 samples from low (*n* = 1) and intermediate (*n* = 10) risk tumours, confirmed by histology were analysed as well. The single low-risk sample was separated from the intermediate risk samples due to reduced lactate, pyruvate and lipids/ lipoproteins and increased citrate, myo-inositol, spermine and fructose. Within these samples, a discrimination was observed due to primary Gleason pattern 4 (present against absent) and was associated with higher levels of lipids/ lipoproteins, lactate, and pyruvate as well as lower levels of citrate, spermine, and myoinositol. These relationships were also observed when the analysis was expanded to all low and intermediate risk patients (determined by biopsy or RP). The models were non predictive when benign samples were considered with risk group combinations and primary Gleason pattern 4 presence.

##### In Situ Magnetic Resonance Spectroscopic Imaging (MRSI) Studies

MRSI allows NMR spectroscopic analysis of prostate tissue in situ, i.e., without taking a biopsy or tissue resection, guided by magnetic resonance imaging. In a first study, Weis et al. aimed at estimating the concentrations of prostate choline (Cho), spermine (Spm) and citrate (Cit) in the benign peripheral zone (PZ) and the benign central gland zone (CG) [65]. Subsequently, the relationship between metabolite concentrations and apparent diffusion coefficient (ADC) in benign prostate tissue was investigated by 3D MRSI. Forty-six patients with biopsy proven PCa were scanned in addition to 10 healthy volunteers. In benign prostate tissues, positive correlations and/or trends were found between Spm, Cho, and Cit concentrations and also between ADC and Cit. Whereas no significant difference in Spm concentration was found between PZ and CG, differences between Cho and Cit concentrations were significant. Moreover, Cho content in PZ was higher than in CG and Cit concentration of benign tissues was found higher in PZ than in CG. Considerable increase of Cho and (Cho + Spm + Cr)/Cit levels and decrease of Cit were found for spectra of malignant tissue. Due to difficulties in quantifying Cr levels, the use of the (Cho + Spm + Cr)/ Cit ratio instead of (Cho + Cr)/Cit or Cho/Cr for PCa detection in CG was proposed.

In a pilot study by Nagarajan et al., the validation of two different nonlinear reconstruction methods, namely maximum entropy (MaxEnt) and total variation (TV), for PCa detection were intended [66]. For that purpose, twenty-two PCa patients were investigated. They observed significantly high mean Cit metabolite ratios in cancerous compared to non-cancerous locations as well as increased levels of Spm, myo-inositol (mI) and decreased levels of glutamate plus glutamine (Glx), but none of the ratios could discriminate between different stages of the disease. Logistic regression analysis and ROC analyses suggested that Cit ratio gives the best predictability (AUC = 94%) in MaxEnt compared to TV. The same authors, in a previous study, investigated magnetic resonance spectroscopy imaging and diffusion-weighted imaging of 41 patients with different pathologically proven different Gleason scores [67]. They confirmed that the ratio (Cho+Cr)/Cit measured in the lesion is positively correlated with the Gleason score, showing an increase of the Cho level and decrease of the Cit level with rising cancer aggressiveness. Furthermore, the study supported that lower apparent diffusion coefficient values were associated with higher Gleason scores. With the same scope, Kobus et al. have assessed tumour aggressiveness by combining the Cho+Cr/Cit and Cho/Cr ratios [68]. In ROC curve analyses, they confirmed the results reported by Nagarajan and colleagues, using either Cho+Cr/Cit ratio or Cho/Cr ratio. They were able to separate low-grade from high-grade tumours with AUC values of 0.70 and 0.74, respectively. The combination of both ratios performed even better (AUC = 0.78), but the improvement was not statistically significant. Wang et al. investigated the usefulness of 1H-MRSI for predicting the proliferative activity of PCa by comparing the (Cho+Cr)/Cit ratio in 38 patients with PCa and 33 patients with BPH [69]. They found a consistent increase of the ratio from the peripheral zone over BPH to PCa.

Kumar et al. attempted to evaluate the incidence of prostate cancer in men with increased PSA level of 4 to 10 ng/mL and a negative MRSI study [70]. Thirty-six patients with negative MRSI findings underwent standard transrectal ultrasound (TRUS) guided sextant biopsy and none was positive. Of the 36 men, 26 completed an 18-month follow-up period. PSA levels increased in four men. A repeat saturation biopsy was benign in three men and PCa was detected (with a Gleason score of 4) in one man 29 months after the initially negative MRSI, leading to an overall predictive value of 96% for a negative MRSI.

#### 3.2.5. Summary

A plethora of PCa biomarker candidates have been evaluated. Candidate biomarkers often differed by specimen type, analytical platform and statistical analysis methods applied, there are nevertheless a number of metabolites reported repeatedly and/or independently validated (Table 5). These metabolites can also provide insight into pathophysiological mechanisms In blood, Mondul et al. [15] and Schmidt et al. [19] have both observed inverse associations of lipids, including glycero-phospholipids with risk of aggressive PCa. Among the nine studies searching for markers for PCa diagnosis, only one study used a test set to validate their biomarkers. Andras and colleagues successfully validated Lysophosphatidylcholine 18:2, homocysteine-inosine, methyladenosine, lipoic acid, hydroxymelatonin, and decanoylcarnitine as diagnostic biomarkers in an independent test set [20]. Interestingly, decanoylcarnitine was also discovered earlier by Giskeødegård and co-workers [52]. In addition, Kumar and colleagues replicated the amino acids alanine and glycine, as well as sarcosine as biomarkers for PCa diagnosis in two independent reports [21,22]. With respect to PCa staging, none of the proposed biomarkers in blood could be validated or replicated consistently. PCa biomarkers for prognosis and therapy prediction warrant further validation.

Urine-based studies reported multiple metabolites to be associated with PCa. Two studies validated their findings using an independent validation set. Fernández-Peralbo et al. validated urea, 7-methylguanine, tyrosine, citrulline and histidine, acetyllysine, acetylhistidine, dimethyllysine and trimethyllysine [35]. However, none of these candidate biomarkers were replicated in the various other studies using urine samples. Zhang et al. compared 30 additional cases to their original control group and reported ureido isobutyric acid, indolylacryloylglycine, acetylvanilalinine and 2-oxoglutarate as consistently associated with PCa [59]. Pérez-Rambla et al. and Struck-Lewicka et al. had glycine in common as a PCa biomarker [37,38]; all other reported metabolites were not replicated. None of the studies that attempted to identify metabolites for PCa staging showed significant results. Preliminary results on PCa risk prediction and PCa recurrence are waiting for further validation.

In tissue, numerous metabolites were found by the different studies. Some metabolites were independently reported by different groups. In regard to PCa diagnosis, uracil [28,42,45,47] and proline [28,44,46,47] were most frequently reported as tissue biomarkers and uracil was independently validated in one study. In addition, there was some evidence for glycine, kynurenine, glycerol-3-phosphate, leucine, choline and citrate as potential diagnostic markers of PCa. For staging purposes, citrate [52,54] along with the polyamines spermine [52,53,54,55] and putrescine [44,55] were the most cited metabolites in tissue. Choline-containing metabolites, especially choline, phosphocholine, and glycerophosphocholine, might be additional important biomarkers for aggressive PCa, although there were contradictory results among different studies. Only a few studies applied metabolomics to find tissue biomarkers to predict biochemical recurrence, and none of the proposed biomarkers was validated neither by additional studies. The ratio of choline, citrate, creatine, myo-inositol is often used for the determination of cancer tissue in the prostate gland by using magnetic resonance spectroscopy imaging (MRSI) There is a single study on seminal fluid [Roberts et al]. Within these samples, a primary Gleason pattern 4 was associated with lower levels of citrate and spermine.

## 4. Discussion

Currently, the paradigm of PCa management would benefit from robustly validated novel biomarkers not only for optimising widely used PSA testing and MRI scans, but also for phenotyping tumour biology before and after treatment. During the last decade, metabolomics is an emerging and promising tool in PCa biomarker development. A plethora of multivariate biomarker sets for different use cases has been studied. The vast majority of these studies report the identification of biomarker candidates with the ability to distinguish malignant from benign prostate tissue with a few studies investigating disease progression and treatment response or tumour recurrence. In general, the reported metabolomic analyses yield high dimensional datasets and the number of analysed metabolites often significantly exceeded the number of available samples. Hence, observed multivariate differences between case and control samples in the datasets might potentially not only be associated with identified biomarkers but also by pre-analytical, technical or statistical artefacts and confounding factors. As powerful machine learning and data mining procedures are particularly suited to find even subtle differences in datasets to enable data discrimination, these techniques may be prone to false-positive associations of arbitrary metabolite combinations with the outcome data, an effect often referred to as “over fitting”. The general heterogeneity of the datasets prevents a precise validation of a biomarker or combination of biomarkers based on the group of samples used for discovery even when utilizing cross-validation procedures. Therefore, as best practice, metabolomics studies should include the validation of the findings using an appropriate set of independent samples useful for the assessment of any clinical effectiveness as a mandatory step.

For the vast majority of reported biomarkers, correcting for multiple testing was rarely applied and replication through use of a clinical independent validation was mostly lacking. Therefore, currently it remains unclear to which extent the reported candidates may represent false positive statistical artefacts. This statistical correction is exemplified by the most commonly cited metabolomics PCa marker, sarcosine. In contrast to the very promising initial reports on sarcosine [28,31,44,71], several groups could not replicate its associations with prostate cancer phenotypes [14,29,32,40]. It remains unclear what the reasons are for these differences in reported findings. In general, the reported studies were comparable in terms of sample sizes. It is striking that the sample size of several dozens or up to 100 cases per group are quite limited compared to the number of detected metabolites per analysis, typically more than 1000 metabolites. This mismatch together with the given biodiversity of samples and cohorts from different continents might lead to false-negative or false-positive findings. Technical characteristics of the different laboratory developed, non-standardized analytical platforms, i.e., liquid chromatography and gas chromatography coupled to mass spectrometry, may also be a further potential reason for the lack of correlation [72]. There are several studies reported here that yielded conflicting results even with consistency of analytical platform [14,29,44]. Additionally, especially for urine based approaches, the differences in findings might well be associated with technical hurdles related to accurately determining the sarcosine/creatine ratio [73]. This fact was also considered by Gamagedara et al., who observed a strong correlation between several urinary marker candidates and urinary creatinine concentrations and concluded that their renal excretion rates might represent a major confounder in urinary studies [34].

Therefore, clinical metabolomics can only overcome its infancy and solve clinical problems when studies for biomarker discovery and validation are carefully designed with appropriate controls in place at the pre-analytical, the analytical and the clinical stage. Sample size is a very crucial element to the analysis. Indeed, the choice of the statistical tests and the performance of the data processing and modelling are dependent on the sample size of datasets. Unfortunately, sample size calculations for these types of studies is not easy. This highlights the importance of collaboration between research groups in terms of biostatistics, standardizing analytical strategies, choosing appropriate clinical references to maximise reproducibility, reliability and robustness of results. Given all these technical and methodological hurdles, there are nevertheless a number of metabolites and pathways repeatedly reported across various technical approaches, cohorts and sample types that appear to play a predominant role in PCa tumour biology, progression and recurrence. Although encouraging, it also demonstrates the enormous challenges faced by metabolomics, which the field is just started to address. Therefore, caution must be taken until a more holistic picture of the strength and weaknesses of novel candidates is available to ensure that they simply are not the most abundant, easy to measure or stable analytes. When clinical metabolomics learns these critical lessons and the field matures, it will benefit patients. However, expectations should not be raised erroneously high, and so key evidence-based standards are essential. When a critical mass of evidence supports a metabolite as having clinical efficacy in initial validation studies, the next stage in the development should be its application in larger prospective studies to demonstrate its clinical utility. In conclusion, the study of the prostate cancer metabolome is still at an early stage, but it may be an important tool for understanding the development and progression of prostate cancer and for developing biomarkers to support its treatment.

## Figures and Tables

**Table 1:** Results of the metabolomics studies conducted on blood samples.

Author	Year	Sample Size	Technique	Use Case	Biomarkers Candidates	Validated?	Statistical Details	Comments
Schmidt [19]	2017	PCa: 1077 Controls: 1077		PCa risk	citrulline	No	Conditional logistic regression	Study population from European Prospective Investigation into Cancer and Nutrition (EPIC) Fasting not required
Huang [16]	2017	*n* = 338 PCa: 72 (T2), 51 (T3), 15 (T4) Controls: 200	LC-MS GC-MS	Staging PCa risk	N-acetyl-3-methylhistidine (T2)lycerophospholipid oleoyl-linoleoyl-GPI (T3)	No	logistic regression *p* = 0.05	Study population from the ATBC Study cohort Overnight fasting serum Time serum collection - Dx avrg = 10 years (range 1–20)
Andras [20]	2017	Training Set: *n* = 59 Validation Set: *n* = 31	HPLC- ESI+ QTOF - MS	Diagnosis	lisophosphatidylcholine 18:2, homocysteine-inosine, methyladenosine, lipoicacid, hydroxymelatonin and decanoilcarnitine	Yes	Mann–Whitney test PLSR-DA ROC analysis	Assessement of the predective value of metabolomic analysis for the presence of PCa at the first systematic biopsy
Dereziński [27]	2017	*n* = 89 PCa: 49 HC: 40	LC-ESI-MS/MS	Diagnosis	methionine, ethanolamine, glutamine, isoleucine, arginine, leucine	No	Mann–Whitney U test Student’s *t*-test Welch’s F test PLS-DAROC analysisv discriminant function analysis	targeted analysis of 32 amino acids in serum urine samples were profiled as well
Kumar [22]	2016	*n* = 210 HC: 65 BPH: 70 PCa: 75	NMR	Diagnosis	HC vs. PCa: alanine, sarcosine, glycine, citrate BPH vs. PCa alanine, sarcosine, glycine, citrate, creatinine HC vs. BPH + PC: aglycine, sarcosine, alanine, creatine, xanthine, and hypoxanthine HC vs. BPH: glycine, xanthine, pyruvate, methylhistidine, and creatinine	Yes	ANOVA Student–Newman–Keuls test DFA ROC analysis	Overnight fasting
Huang [17]	2016	PCa: 380 controls: 380	UPLC-MS GC-MS	PCa risk	pyroglutamine, gamma-glutamylphenylalanine, phenylpyruvate, N-acetylcitrulline, and stearoylcarnitine	No	Conditional logistic regression *p* = 0.000072	Study population from the Prostate, Lung, Colorectal and Ovarian Cancer Screening Trial (PLCO) Fasting not required
Giskeødegård [23]	2015	*n* = 50 PCa: 29 BPH: 21	MRS GC-MS	Diagnosis	decanoylcarnitine (c10), tetradecenoylcarnitine (c14:1), octanoylcarnitine (c8), dimethylsulfone, phenylalanine, lysine, phosphatidylcholine diacyl C34:4, lipid signals -(CH2)n-CH2-CH2-CO	No	PCA (no discrimination) OPLS-DA and OPLS Wilcoxon rank sum testing (*p* ≤ 0.05) ROC analyses	Fasting serum and plasma samples Missing data for some variables were replaced by estimated values using a built-in data imputation algorithm
Mondul [15]	2015	*n* = 400 PCa: 200 (100 aggressive) Controls: 200	UPLC-MS GC-MS	PCa risk	Inositol-1-phosphate oleoyl-linoleoylglycerophosphoinositol, 1-stearoylglycerophosphoglycerol, stearate and docosadienoate. Both alpha-ketoglutarate and citrate were associated with aggressivedisease risk as were elevated thyroxine and trimethylamine oxide	No	Conditional logistic regression Threshold for statistical significance *p* = 0.003 in the main analysis	Study population: from Alpha-Tocopherol, Beta-Carotene Cancer Prevention Study cohort Fasting serum collected up to 20 years prior to case diagnoses Missing values were assigned the minimum nonmissing value.
Kumar [21]	2015	*n* = 102 PCa: 70 (40 low grade PCa, 30 high grade PCa) HC: 32	NMR	Diagnosis Staging	HC vs. PCa: alanine, pyruvate, glycine, sarcosine low grade PCa vs. high grade PCa: alanine, pyruvate, and glycine	No	Unsupervised PCA supervised OPLS-DA ANOVA Student–Newman–Keuls test ROC analysis	Fasting serum samples
Mondul [14]	2014	PCa: 74 Controls: 74	UPLC-MS GC-MS	PCa risk	1-stearoylglycerol Glycerolalpha-ketoglutarate	No	Logistic regression threshold for statistical significance: 0.000119	Study population from the Alpha-Tocopherol, Beta-Carotene Cancer Prevention (ATBC)study Overnight fasting serum
Zang [26]	2014	*n* = 114 PCa: 64 Controls: 50	UPLC-LS/MS	Diagnosis	fatty acids, amino acids, lysophospholipids, and bile acids	No	SVM PCA significance level 0.05	40 discriminant metabolites are found Only the top ranking ones are presented here
Huang [30]	2014	*n* = 72 newly diagnosed PCa: 18 HC: 18 good ADT responders: 18 poor ADT responders: 18	LC-MS	Therapy prediction	deoxycholic acid (DCA), glycochenodeoxycholate (GCDC), L-tryptophan, docosapentaenoic acid (DPA), arachidonic acid, deoxycytidine triphosphate, and pyridinoline	No	PLS-DA and OPLS ANOVA Statistical significance *p* = 0.05	Fasting serum (overnight fast)
de Vogel [18]	2014	PCa: 3000 Controls: 3000	LC-MSGC-MS	PCa risk	Sarcosine; glycine	No	condiotional logistic regression	Study population within the JANUS cohort Fasting status: unknown
Fan [24]	2010	*n* = 56 PCa: 42 (20 GS5, 22 GS7) BPH: 14	NMR	Diagnosis	glutamate and formate	No	ANOVA Random Forests	Article focusing on proteomics by applying RF to 2D-DIGE data NMR data were also presented but with much lower predictive performance
Osl [25]	2008	*n* = 320 Controls: 114 PCa: 206	FIA-MS/MS LC-MS/MS	Diagnosis Staging	Diagnosis: PC a C16:0, PC a C18:0, Serotonin, Aspartate, OrnithineStaging: No reliable biomarkers	No	Associative Voting algorithm Logistic regression	Study population: Men participation in PCa screening One simple rejected
Stabler [31]	2011	*n* = 58 patients after radical prostatectomy recurrent free: 30 recurrent: 28	GC-MS	Prognosis	homocysteine, cystathionine, cysteine	No	Wilcoxon rank sum test Logistic regression Likelihood ratio ROC analysis Kaplan Meier plots Cox proportional hazard regressio models	analysed both serum and urine samples targeted analysis of sarcosine, dimethylglycine, methionine, homocysteine, vystathionine, cysteine, methylmalonic acid, methylcitrate
Thysell [29]	2010	PCa with metastases: 7 PCa w/o metastases: 6 benign: 17	GC-TOFMS	Staging	pseudouridine, creatinine, glucose, glutamate, taurine, phenylalanine, stearate	No	OPLS-DA Mann–Whitney U-test	main work was done on tissue extracts from fresh-frozen biopsies of bone metastases and from biopsies of primary PCa and benign prostate all patients were selected to have hihg-risk tumours (i.e., presence of bone metastases, locally advanced tumour or poorly differnitated cancer)
Sreekumar [28]	2009	*n* = 42 tissue samples benign adjacent: 16 localized PCa: 12 metastatic PCa: 14	UHPLC-MS/MS GC-MS	Diagnosis	blood metabolites not reported	No	Wilcoxon rank-sum test	study analyzed tissue, blood, and urine samples but focused on tissue data only

ADT: androgen deprivation therapy; ANOVA: one-way analysis of variance; avrg: average; BPH: benign prostate hyperplasia; DFA: discriminant function analysis; Dx: diagnosis; ESI: electrospray ionization; FIA: flow injection analysis; GS: Gleason score; GC: gas chromatography; HC: healthy control; (HP/UP)LC: (high performance/ultra performance) liquid chromatography; MRS: magnetic resonance spectroscopy; MS: mass spectrometry; MS/MS tandem mass spectrometry; NMR: nuclear magnetic resonance; OPLS-DA: orthogonal projections to latent structures-discriminant analysis; (Q)TOF: (quadrupole) time of flight; PCa: prostate cancer; PCA: principal component analysis; PLS(R)-DA: partial least squares (regression)-discriminant analysis; RF: random forests; ROC: receiver-operating characteristic; SVM: support vector machine; T2,T3,T4: tumor stages according to TNM classification.

**Table 2:** Results of the metabolomics studies conducted on urine samples.

Author	Year	Sample Size	Technique	Use Case	Biomarkers Candidates	Validated?	Statistical Details	Comments
Pérez-Rambla [37]	2017	*n* = 115 PCa: 64B BPH: 51	1H-NMR	Diagnosis	branched-chain amino acids (BCAA), glutamate and pseudouridine glycine, dimethylglycine, fumarate and 4-imidazole-acetate.	No	PCA analysis OPLS-DA *p* = 0.01	
Gkotsos [33]	2017	PCa: 32 elevated PSA: 101 HC: 15	UPLC-MS/MS	Diagnosis Prognosis	kynurenic acid	No	ROC analysis *p* = 0.05	Ultrasonographically-guided prostatic biopsy collected before and after prostatic massage for the 101 patients Urinary concentrations of metabolites were not normalized to urinary creatinine The control group was recruited only from individuals presenting for other etiologies and these patients usually are much younger than those presenting prostate cancer.
Fernández- Peralbo [35]	2016	*n* = 104 PCa: 62 HC: 42	LC-QTOF	Diagnosis	28 significant metabolites	No	PLS-DA *t*-test with Benjamini–Hochberg false discovery rate *p* = 0.05	Morning urine samples The controls are negative biopsy individualsCases are patients with significant PCa confirmed by prostate biopsy MSTUS was selected for normalization ofurine samples
Tanzeela [36]	2015	PCa: 59 HC: 43	GC/MS	Diagnosis	2,6-dimethyl-7-octen-2-ol, pentanal, 3-octanone, and 2-octanone	No	RF LDA	Urine samples were obtained at different times of the day samples were classified as prostate cancer or controls after pathological examination of the biopsy specimens
Struck-Lewicka [38]	2015	PCa: 32 HC: 32	HPLC-TOF/MS GC-QqQ/MS	Diagnosis	metabolites involved in biochemical pathways like AA, purine and glucose metabolism as well as urea and TCA cycle	No	PCA analysis PLS-DA *p* = 0.05	
Zhang [32]	2013		LC-HRMS HILICRP	Diagnosis	ureido isobutyric acid, indolylacryloyglycine, acetylvanilalinine and 2-oxoglutarate	Yes (PCa:30)	OPLS-DA ROC analysis *p* = 0.05	Urine samples were stored at $-30{\;}^{\circ }C$ Three independent normalisation methodsTesting against a new cohort of patients.
Gamagedara [34]	2012	PCa: 63 HC: 68	LC-MS/MS	Diagnosis Prognosis	proline, kynurenine, uraciland glycerol-3-phosphate	No	Linear regression PCA analysis CART *p* = 0.05.	Validation study
Wu [40]	2011	PCa: 20 BPH: 8 HC: 20	ID GC/MS MAD	Diagnosis	PCa vs. HC: Propenoic acid, Pyrimidine, Dihyroxybutanoic acid, Creatinine, Purine, Purine, Glucopyranoside, Ribofuranoside, Xylonic acid, Xylopyranose PCa vs. BPH: Dihyroxybutanoic acid, Pyrimidine, Xylonic acid, Xylopyranose, Ribofuranoside	No	PCA analysis ROC analysis *p* = 0.05	
Cao [41]	2010	PCa: 86 HC: 45	LC-MS	Diagnosis	Sarcosine	No	Logistic regression ROC analysis *p* = 0.05	First voided urines after digital rectalexamination (DRE)
Kosti [39]	2010	PCa: 77 HC: 77 Biopy controls: 37	LC-MS	Predection	16-ketoestradiol 17-epiestriol	No	Logistic regression	The biopsy controls had elevated PSA due to BPH (*n* = 27) or other urologic conditions (10)
Dereziński [27]	2017	PCa: 49 HC: 40	LC-ESI-MS/MS	Diagnosis	γ-amino-n-butyric acid, phosphoethanolamine, ethanolamine, homocitrulline, arginine, δ-hydroxylysine and asparagine	No	ROC analysis PLS-DA *p* = 0.05	Evaluation of free amino acid profiles in both urine and serum samples from the same patiens Controls recruited among men subjected to the routine periodic medical examination Sample collection period over 3 months
Stabler [31]	2011	PCa: 54	GC-MS	Reccurence	sarcosine and cysteine	No	Logistic regression *p* = 0.007	Study conducted on both urine and serum Subjestcs were divided into 2 groups: - Cases who developed biochemical recurrnce within 2 years - Controls who remained recurrence-free after 5 years.

BPH: benign prostate hyperplasia; CART: classification and regression trees; ESI: electrospray ionization; GC: gas chromatography; HC: healthy control; (HP/UP/HILICP)LC: (high performance/ultra performance/hydrophilic interaction and reversed phase) liquid chromatography; MS: mass spectrometry; MS/MS tandem mass spectrometry; NMR: nuclear magnetic resonance; OPLS-DA: orthogonal projections to latent structures-discriminant analysis; QqQ/MS: triple quadrupole mass spectrometry; (Q)TOF: (quadrupole) time of flight; PCa: prostate cancer; PCA: principal component analysis; PLS(R)-DA: partial least squares (regression)-discriminant analysis; RF: random forests; ROC: receiver-operating characteristic.

**Table 3:** Results of the metabolomics studies conducted on tissue samples.

Author	Year	Sample Size	Technique	Use Case	Biomarkers Candidates	Validated?	Statistical Details	Comments
Wang [50]	2017	*n* = 3 subjects Cancer tissue: 3 Benign adjacent tissue: 3	MALDI-FTICR-MS	Diagnosis	differential metabolites were not mentioned	No	students *t*-test	coated tissue slice
Huan [42]	2016	Training set: *n* = 16 Pca: 13; Benign: 12 Validation set 1: *n* = 18 Pca: 19; Benign: 17 Validation set 2: *n* = 12 Pca: 12; benign: 12	LC QTOF-MS	Diagnosis	adenosine monophosphate, spermidine, uracil, ophthalmic acid + HPO3, 2,3-diaminopropionic acid + HPO3 + 2 unknown metabolites = putative identification	Yes	OPLS-DA volcano plot	Tissue extracts from core biopsies after prostatectomy
McDunn [44]	2013	Pca: 331 Benign: 178 (matched to 178 of the Pca samples)	UHPLC-MS/MS GC-MS	Staging	aggressive Pca:ADP, Glucose, 6-sialyl-N-actyllyctosamine, 2-hydroxypalmitate, 5,6 dihydrouracil, choline, fumarate, kynurenine, phophate, 2-hydrxoystearate, Ac-SDKP, choline phosphate, glycerol-3-phophate, n-acetylaspartate Gleason pattern progression:proline, malate, ADP-ribose, 6-sialyl-N-acetyllactosamineextracapsular extension:NAD+, N-acetylaspartate, putrescine, Glucose Tumor spread (regional lymph nodes/seminal vesicles):choline phosphate, Glycerol3-phophate, putrescine, 6-sialyl-N-acetyllactosamine	No	paired *t*-testWilcoxon test linear regression	Tissue extract from OCT embedded tissue from prostatectomies
Jung [47]	2013	*n* = 95 matched cancer and benign adjacent tissue	LC-MS/MS GC-MS	Diagnosis Prognosis Biological recurrence	Diagnosis of Pca:2-hdroxybehenic acid, crebronic acid, tricosanoic acid, glycerophophoethanolamine, isopentenyl pyrophosphate, 7-methylguanine, 2-aminoadipic acid, gluconic acid, maltotriose, tricosanoic acid Prediction of biological recurrence risk:2-aminoadipic acid, gluconic acid, maltotriose	No	Wilcoxon paired test ROC analysis logistic regression Kaplan–Meier curves univariate and multivariate Cox regression	tissue extracts of punch biopsy from cryosections after prostatectomy
Brown [51]	2012	*n* = 8 matched cancer and benign adjacent tissue	UHPLC-MS/MS GC-MS	Diagnosis	> 40 metabolites not specified further	No	Welch’s two sample *t*-test hierachical clustering	tissue extracts from core biopsy after prostatectomy
Selnæs [53]	2012	*n* = 13 subjects 40 tissue samples	HR-MAS-1H-MRS	Staging	CCS/C ratio (choline+creatine+spermine over citrate)	No	Spearman’s rank correlation Jonchheere– Terpstra test	intact tissue from needle biopsy after prostatectomy
Maxeiner [60]	2010	Pca with biological recurrence: 16 Pca without biological recurrence: 32	HR-MAS-1H-MRS	Prognosis	spermine, glutamine, myo-inositol, phophoryl choline, scylloinositol, glutamate	Yes	PCA student’s *t*-test Canonical analysis ANOVA ROC analysis	intact tissue from needle biopsy after prostatectomy;training and test set with identical case group but distinct control groups
Sreekumar [28]	2009	*n* = 42 tissue samples benign adjacent: 16 localized Pca: 12 metastatic Pca: 14	UHPLC-MS/MS GC-MS	Diagnosis	sarcosine, uracil, kynurenine, glycerol-3-phophate, leucine, proline	Yes	Wilcoxon rank-sum test	tissue extracts from biopsy samplesonly sarcosine was analyzed in validation set
Cacciatore [49]	2017	matched benign and Pca samples Training set: *n* = 8 Validation set: *n* = 4	UHPLC-MS/MS GC-MS	Diagnosis	32 metabolites reported; biomarkers included in the model not specified	Yes	Hierarchical clustering OSC-PLS	tissue extracts from tissue section after prostatectomyalso compared OCT-embedded and FFPE tissue as biospecimen
Sandsmark [54]	2017	*n* = 41 subjects Pca: 95 benign adjacent: 34	HR-MAS-1H-MRS	Diagnosis	Pca with high vs. Pca with low/intermediate NCWP-EMT score: spermine and citratePca with low vs. Pca with high NCWP-EMT score taurine, phosphoethanolamine	No	*t*-test	intact tissue from prostatectomiesmain focus: alterations in non-canonical WNT signaling pathway (NCWP) and EMT in Pcajoint gene expression and metabolomic analyses; targeted analysis of 23 metabolites; metabolomics was performed only on the main cohort
Hansen [55]	2016	*n* = 41 subjects Pca: 95 benign adjacent: 34	HR-MAS MRS	Staging	citrate, spermine (correlated with presence ERG translocation)	Yes	unsupervised multivariate analysis PLS-DA	gene expression analysis and TMPRSS2-ERG as marker for disease aggressiveness;intact tissue from tissue slices collected from prostatectomies; analyzed metabolic alterations in PCA patients positive for TMPRSS2-ERG/high ERG gene fusion; targeted analysis of 23 metabolites
Meller [57]	2016	*n* = 106 subjects matched cancer and benign adjacent tissue ERG-positive Pca: 27 ERG-negative Pca: 23	GC-MS LC-MS	Staging Prognosis Biological recurrence	Gleason score:pantothenic acid, maltose, fructose-6-phosphate, gluconic acid, cholesterol ERG status:maltotriose, gluconic acid, citrate, cis-aconitate, spermine, putrescine, cerebronic acid, 2-hydroxybehenic acid, tricosanoic acid, Biological relapse:tyrosine and tryptophan	No	ANOVA PCA	tissue extracts of punch biopsy from cryosections after prostatectomy
Ren [43]	2016	Training set:25 paired PCa and adjacent benign Validation set:51 paired Pca and adjacent benign 16 BPH	LC-MS	Diagnosis	sphingosine, citicoline, choline, pantothenic acid, carnitine C4-OH, GPC, NAD, phenylacetyl-glutamine, carnitine C14:2	Yes	PCA PLS-DA Signrank Wilcoxon signed rank two-sides test (biomarker analysis)	joint transciptomics and metabolomics to identify altered metabolic pathways in PCA tissue;tissue extracts from prostatectomies
Liu [46]	2015	*n* = 42 tissue samples benign adjacent: 16 localized Pca: 12 metastatic Pca: 14	n.a.	Diagnosis	Proline, Cholesterol, sarcosine, spermidine, spermine, Putrescine, 4-Acetamidobutanoate	Yes	DRW-GM + logistic regression	joint analysis of genomic and metabolomic data and pathway topology using directed random walk on a global gene-metabolite pathway graph;used dataset established by Sreekumar et al. 2009
Priolo [48]	2014	Discovery set: Pca: 61; benign: 25 Validation set: Pca: 40; benign: 16	UHPLC-MS/MS GC-MS	Diagnosis Tumour subtyping	MYC-driven Pca:Oleic acid, arachidonic acid, docosahexaenoic acidsAKT1-driven Pca:2-aminoadipic acid, creatine	Yes	Mann–Whitney test	metabolomic profiling of tumors driven by MYC and AKT1 oncogenes;extracts of frozen tissue from prostatectomy;metabolomic profiling in cell lines, mice and human tissue; validation of selected markers in human tissue samples
Keshari [58]	2011	*n* = 49 tissue samples high-grade Pca: 13 low-grade Pca: 22 benign: 14	1-D and 2-D HR-MAS Spectroscopy	Staging	Benign vs. Pca tissue: choline, phosphocholine, glycerophosphocholine, phosphoethanolamine, glycerophosphoethanolamine, citrate, polyamineslow-grade vs. high-grade Pca:phosphocholine, glycerophosphocholine	No	Student’s *t*-test	intact tissue from core biopsies after prostatectomy;targeted analysis of phospholipid metabolites
Shuster [45]	2011	Pca: 14 benign: 14	GC-MSLC-MS/MS	Diagnosis	cysteine, dihomo-linoleate, docosapentaenoate, N-acetylaspartate, N-acetylglucosamine, uracil, xanthine, and 1-stearoylglycerophophoinositol;uracil, kynurenine, glycerol-3-phosphate, leucine, proline; choline, lactate, alanine citrate, putrescine, spermidine, spermine	No	matched paired *t*-test	tissue extracts from needle biopsies after prostatectomy;description of the mPREF methodreplicated metabolites previously published by Sreekumar et al. 2009 and various in vivo studies
Zhang [59]	2014	untreated patients: benign: 58; indolent Pca: 5; aggressive Pca: 8 radiation-treated patients: benign: 32; indolent Pca: 7 (relapse); aggressive Pca: 12 (relapse)	1-D and 2-D HR-MAS Spectroscopy	Staging Diagnosis	aggressive vs. indolent Pca:choline, phosphocholine, glycerophosphocholine, [choline + phosphocholine + glycerophosphocholine] to creatine ratio, (lactate; only in untreated)benign vs. Pca (untreated):citrate, polyamines, lactate, glutamate, alanine	No	linear mixed-effects model Wilcoxon Rank Sum Test Kruskal–Wallis Test	intact tissue from core biopsies
Giskeødegård [52]	2013	*n* = 158 tissue samples from 48 subjects benign: 47v low-grade Pca: 30 high-grade Pca: 81	HR-MAS 1H MRS	Staging	Pca vs. benign: citrate, taurine, creatine, glycerohpophocholine, phosphocholine, choline, glycinelow-grade vs. high-grade Pca:spermine, citrate, CCP/C ratio	No	Linear mixed models PLS-DA models	intact tissue from biopsies
Jentzmik [56]	2011	*n* = 92 Matched PCa and adjacent benign tissue	GC-MS	Staging	sarcosine	No	Mann–Whitney U test Wilcoxon test Spearman rank correlation Kaplan–Meier curve Cox proportional hazards regression analysis log rank test ROC analysis	target analysis of sarcosine as biomarker for disease progression tissue extracts from punch biopsies of tissue sections collected after prostatectomy
Thysell [29]	2010	Discovery set: bone metastases: 14 (hormone-naive Pca: 7; CRPC 7) adjacent normal bone: 10 Validation set:bone metastases: 13(6 Pca, 7 other cancers) normal bone: 11 Primary tumour: with metastases: 7 w/o metastases: 6benign: 17	GC-TOFMS	Staging	Bone tissue: metastases vs. normal: Cholesterol, myo-inositol-1-phosphate, citrate, fumarate, glycerol-3-phosphate, amino aicds Primary tumour: metastatic PCa vs. benign tissue and non-metastatic PCa:malate, dehydroascrobic acid, urea, hypoxanthine, asparagine, threonine, fumarate, linoleic acid	Yes	OPLS-DA Mann–Whitney U-test	tissue extracts from fresh-frozen biopsies of bone metastases and from biopsies of primary Pca and benign prostateall patients were selected to have hihg-risk tumours (i.e., presence of bone metastases, locally advanced tumour or poorly differnitated cancer)validation set available only for bone metastatic tissueblood plasma samples from men who underwent prostate biopsies were also analyzed

ADT: androgen deprivation therapy; ANOVA: one-way analysis of variance; BPH: benign prostate hyperplasia; GC: gas chromatography; (HP/UP)LC: (high performance/ultra performance) liquid chromatography; MALDI-FTICR: matrix-assisted laser desorption/ionization Fourier-transform ion cyclotron resonance mass spectrometry imaging; MRS: magnetic resonance spectroscopy; MS: mass spectrometry; MS/MS tandem mass spectrometry; (HR-MAS) NMR: (high resolution - magic angle spinning) nuclear magnetic resonance; OPLS-DA: orthogonal projections to latent structures-discriminant analysis; (Q)TOF: (quadrupole) time of flight; PCa: prostate cancer; PCA: principal component analysis; PLS(R)-DA: partial least squares (regression)-discriminant analysis; ROC: receiver-operating characteristic.

**Table 4:** Results of the metabolomics studies conducted on other sample types.

Author	Year	Sample Size	Technique	Use Case	Biomarkers Candidates	Validated?	Statistical Details	Comments
Clos-Garcia [61]	2018	PCa: 31 BPH: 14	UHPLC-MS	Diagnosis Staging Prognosis	phosphathidylcholines, acyl carnitines, citrate and kynurenine steroid hormone, 3beta-hydroxyandros-5-en-17- one-3-sulphate (dehydroepiandrosterone sulphate)	NO	ROC analysis PCA PLS-DA OPLS	Sample type: Urine extracellular vesicles (EVs)
Roberts [63]	2017	*n* = 151 PCa: 9880 (initially diagnosed) 18 (diagnosed during follow-up period)	NMR	Risk prediction	lipids/lipoproteins (PC1)choline phosphocholinecitrate Fructose and spermine	NO	PCA PLS	Sample type: Seminal plasma Time sample collection between January 2007 and February 2013 Samples obtained prior to or at least 1 month after prostate biopsy, prior to commencement of any treatment No other specifications were provided to patients for sample collection process Glucose signals were excluded from the spectra
Puhka	2017	PCA: 3 HC: 3	UPLC-MS	Diagnosis	glucuronate, D-ribose 5-phosphate and isobutyryl-L-carnitine	NO		Sample type: urinary and platelet EVs Urine samples and matched plasma samples were collected 0–3 days before and 5–6 weeks after the prostatectomy Control samples were from healthy <35 year-old mennormalization to EV-derived factors or with metabolite ratios, and not from the original urine samples.
Weis	2016	PCa: 46 HC: 4	3D Proton MRSI	Diagnosis	choline, spermine and citrate ratios	NO		concentration referenced to water
Nagarajan [66]	2015	PCa: 22	EPSI 2D-JPRESS (4D) EP-JRESI	Diagnosis	choline, spermine, citrate, myo-inositol and glutamate plus glutamine (Glx)	NO	logistic regression analysis ROC analysis	Prostate cancer was histopathologically confirmed after RP
Nagarajan [67]	2012	PCa: 41 GS 3+3 (*n* = 12) GS 3+4 (*n* = 20) GS 3+4 (*n* = 9)	MRSI DWI	Staging	choline, creatine and citrate ratios	NO	ROC analysis	At least 6 weeks time period between biopsy and MRI
Kobus [68]	2011	PCa: 43	MR MRSI	Staging	choline, creatine and citrate ratios	NO		3 T
Wang [69]	2008	PCa: 33 BPH: 33	1H-MRSI	Diagnosis	choline, creatine and citrate ratios	NO		1.5 T
Kumar [70]	2008	*n* = 155	MRSI	Diagnosis	choline, creatine and citrate ratios	NO		1.5 T TRUS-guided prostate biopsy within 1 week after the MRSI

BPH: benign prostate hyperplasia; DWI: diffusion-weighted imaging ; EPSI:echo-planar spectroscopic imaging; EP-JRESI: echo-planar J-resolved spectroscopic imaging; GC: gas chromatography; GS: Gleason score; (UHP/UP)LC: (ultra high performance/ultra performance) liquid chromatography; JPRESS: J resolved spectroscopic sequence; MRS: magnetic resonance spectroscopy; MS: mass spectrometry; MS/MS tandem mass spectrometry; NMR: nuclear magnetic resonance; OPLS-DA: orthogonal projections to latent structures-discriminant analysis; PCa: prostate cancer; PCA: principal component analysis; PLS(R)-DA: partial least squares (regression)-discriminant analysis; ROC: receiver-operating characteristic; T: Tesla; TRUS: transrectal ultrasound.

**Table 5:** Most significant metabolites reported over all the selected studies.

	Risk Prediction	Diagnosis	Prognosis	Recurrence
citrate	Mondul et al. 2015 [15] (B)	Kumar et al. 2016 [22]) (B)	Giskeødegård et al. 2013 [52] (T)	
	Huang et al. 2017 [16] (B)	Weis et al. 2017 [65] (O)	Sandsmark et al. 2017 [54] (T)	
	Roberts et al. 2017 [63] (O)	Nagarajan et al. 2015 [67] (O)	Selnæs et al. 2013 [53] (T)	
		Wang et al. 2010 [69] (O)	Hansen et al. 2016 [55] (T)	
		Zhang et al. 2014 [59] (T)	Meller et al. 2016 [57] (T)	
			Keshari et al. 2011 [58] (T)	
			Thysell et al. 2010 [29] (T)	
glycine	de Vogel et al. 2014 [18]	Kumar et al. 2015 [21] (B)	Hansen et al. 2016 [55]	
		Kumar et al. 2016 [22] (B)		
		Pérez-Rambla et al. 2017 [37] (U)		
		Struck-Lewicka et al. 2015 [38] (U)		
		Liu et al. 2015 [46] (T)		
		Jung et al. 2013 [47] (T)		
		Giskeødegård et al. 2013 [52] (T)		
glycerol-3-phosphate		Andras et al. 2017 [20] (B)	McDunn et al. 2013 [44] (T)	
		Sreekumar et al. 2009 [28] (T)	Thysell et al. 2010 [29] (T)	
glycerophosphocholine		Andras et al. 2017 [20] (B)	Giskeødegård et al. 2013 [52] (T)	
			Keshari et al. 2011 [58] (T)	
			Zhang et al. 2014 [59] (T)	
alanine		Kumar et al. 2015 [21] (B)	Kumar et al. 2015 [21] (B)	
		Kumar et al. 2016 [22] (B)	Zhang et al. 2014 [59] (T)	
		Struck-Lewicka et al. 2015 [38] (U)		
		Shuster et al. 2011 [45] (T)		
		Jung et al. 2013 [47] (T)		
		Zhang et al. 2014 [59] (T)		
choline		Ren et al. 2016 [43]	Selnæs et al. 2013 [53] (T)	
		Shuster et al. 2011 [45]	Zhang et al. 2014 [59] (T)	
		Giskeødegård et al. 2013 [52] (T)	Nagarajan et al. 2015 [67] (O)	
		Keshari et al. 2011 [58] (T)	Kobuscet al. 2011 [68] (O)	
		Zhang et al. 2014 [59] (T)		
		Weis et al. 2017 [65] (O)		
		Wang et al. 2010 [69] (O)		
phosphocholine		Giskeødegård et al. 2013 [52] (T)	Zhang et al. 2014 [59] (T)	
		Keshari et al. 2011 [58] (T)		
		Zhang et al. 2014 [59] (T)		
glycerophosphocholine		Andras et al. 2017 [20] (B)	Zhang et al. 2014 [59] (T)	
		Giskeødegård et al. 2013 [52] (T)		
		Keshari et al. 2011 [58] (T)		
uracil		Huan et al. 2016 [42] (T)		
		Sreekumar et al. 2009 [28] (T)		
		Shuster et al. 2011 [45] (T)		
		Jung et al. 2013 [47] (T)		
		McDunn et al. 2013 [44] (T)		
proline		Dereziński et al. 2017 [27] (B)	Sreekumar et al. 2009 [28] (T)	
		Jung et al. 2013 [47] (T)	Liu et al. 2015 [46] (T)	
		McDunn et al. 2013 [44] (T)		
histidine		Fernández-Peralbo et al. 2016 [35] (U)		
		Jung et al. 2013 [47] (T)		
spermine	Roberts et al. 2017 [63] (O)	Nagarajan et al. 2015 (O)	Liu et al. 2015 [46] (T)	
		Weis et al. 2017 [65] (O)	Shuster et al. 2011 [45] (T)	
			Giskeødegård et al. 2013 [52] (T)	
			Selnæs et al. 2013 [53] (T)	
			Sandsmark et al. 2017 [54] (T)	
			Hansen et al. 2016 [55] (T)	
			Meller et al. 2016 [57] (T)	
			Maxeiner et al. 2010 [ 60] (T)	
2-aminoadipic acid		Jung et al. 2013 [47] (T)		
		Shuster et al. 2011 [45] (T)		
		Sreekumar et al. 2009 [28] (T)		
		Priolo et al. 2014 [48] (T)		

B: Blood, U: Urine, T: Tissue, O: Other.

## Abbreviations

The following abbreviations are used in this manuscript:

PCa	Prostate cancer
BPH	Benign prstate hyperplasia
DRE	Digital rectal exam
GS	Gleason score
HC	Healthy controls
NEM	No evidence of malignancy
PSA	Prostate specific antigen
TNM	Tumour-lymphnode-metastasis classification
SP	Seminal plasma
EV	Extracellular vesicles
ADT	Androgen deprivation therapy
AJCC	American Joint Committee on Cancer
CRPC	Castration-resistant prostate cancer
PRISMA	Preferred Reporting Items for Systematic Reviews and Meta-Analyses
MS	Mass spectrometry
GC-MS	Gas chromatography mass spectrometry
MRIS	Magnetic resonance imaging spectroscopy
NMR	Nuclear magnetic resonance
LC-MS	Liquid chromatography mass spectrometry
LC-QTOF-MS	Liquid chromatography quadrupole time-of-flight mass spectrometry
GC-TOF-MS	Gas chromatography time-of-flight mass spectrometry
HPLC-ESI+QTOF-sMS	High performance liquid chromatography electrospray ionization quadrupole time-of-flight mass spectrometry
UHPLC-MS	Ultra high performance liquid chromatography mass spectrometry
HR-MAS-1H-MRS	High resolution magic angle spinning proton magnetic resonance spectroscopy
LC-ESI-MS	Liquid chromatography electrospray ionization mass spectrometry
PCA	Principal component analysis
PLS-DA	Partial least squares-discriminant analysis
RF	Random forest
OSC-PLS	Orthogonal signal correction for partial least square analysis
OPLS-DA	Orthogonal projections to latent structures-discriminant analysis
ROC	Receiver operator characteristics
SVM	Support vector machine
VIP	Variable importance of projection
LDA	Linear discrimination analysis
DFA	Discriminant function analysis
ANOVA	One-way analysis of variance
AUC	Area under the curve
DRW-GM	Directed random walk on gene metabolite pathway graph
(CCP/C)	Choline+creatine+polyamines over citrate
Cho	Choline
Cit	Citrate
Cr	Creatinine
GPC	Glycerophosphocholine
GPE	Glycerophospho-ethanolamine
LCFA	Long chain fatty acid
mI	Myo-inositol
NAD	Nicotinamide adenine dinucleotide (NAD+)
PC	Phosphocholine
PE	Phosphoethanolamine
Spm	Spermine
TMAO	Trimethylamine N-oxide
